# Dynamic evolution and spatial difference of public health service supply in economically developed provinces of China: typical evidence from Guangdong Province

**DOI:** 10.1186/s12913-023-10444-4

**Published:** 2024-01-04

**Authors:** Yan-Ting He, Yue-Chi Zhang, Rang-Ke Wu, Wen Huang, Ruo-Nan Wang, Luo-Xuan He, Bei Li, Yi-Li Zhang

**Affiliations:** 1 School of Health Management, Southern Medical University, Guangzhou510515, China; 2 School of Social & Political Sciences, College of Social Sciences, University of Glasgow, Glasgow, UK; 3 School of Foreign Studies, Southern Medical University, Guangzhou510515, China; 4 The Fifth Affiliate Hospital of Southern Medical University, Guangzhou510515, China

**Keywords:** Public health services, Dynamic evolution, Spatial differences, Standard deviation ellipse method, Kernel density estimation, Markov chain, Dagum Gini coefficient, Panel regression model

## Abstract

**Objective:**

The outbreak of the COVID-19 pandemic has drawn attention from all sectors of society to the level of public health services. This study aims to investigate the level of public health service supply in the four major regions of Guangdong Province, providing a basis for optimizing health resource allocation.

**Methods:**

This article uses the entropy method and panel data of 21 prefecture-level cities in Guangdong Province from 2005 to 2021 to construct the evaluation index system of public health service supply and calculate its supply index. On this basis, the standard deviation ellipse method, kernel density estimation, and Markov chain are used to analyze the spatiotemporal evolution trend of the public health service supply level in Guangdong Province. The Dagum Gini coefficient and panel regression model are further used to analyze the relative differences and the key influencing factors of difference formation. Finally, the threshold effect model is used to explore the action mechanism of the key factors.

**Results:**

Overall, the level of public health service supply in Guangdong Province is on an upward trend. Among them, polarization and gradient effects are observed in the Pearl River Delta and Eastern Guangdong regions; the balance of public health service supply in Western Guangdong and Northern Mountainous areas has improved. During the observation period, the level of public health services in Guangdong Province shifted towards a higher level with a smaller probability of leapfrogging transition, and regions with a high level of supply demonstrated a positive spillover effect. The overall difference, intra-regional difference and inter-regional difference in the level of public health service supply in Guangdong Province during the observation period showed different evolutionary trends, and spatial differences still exist. These differences are more significantly positively affected by factors such as the level of regional economic development, the degree of fiscal decentralization, and the urbanization rate. Under different economic development threshold values, the degree of fiscal decentralization and urbanization rate both have a double threshold effect on the role of public health service supply level.

**Conclusion:**

The overall level of public health service supply in Guangdong Province has improved, but spatial differences still exist. Key factors influencing these differences include the level of regional economic development, the degree of fiscal decentralization, and the urbanization rate, all of which exhibit threshold effects. It is suggested that, in view of the actual situation of each region, efforts should be made to build and maintain their own advantages, enhance the spatial linkage of public health service supply, and consider the threshold effects of key factors in order to optimize the allocation of health resources.

**Supplementary Information:**

The online version contains supplementary material available at 10.1186/s12913-023-10444-4.

## Introduction

Health relates comprehensively to the public health service system built by governments. For decades, the Chinese government has been weaving a public health protection net for the masses based on their governing philosophy of “People, The First”. With this net, the Chinese government has solved the basic health problems of one fourth of the world’s population effectively and efficiently, pushing the grand plan of a Healthy China in progress [1]. In addition to meeting the basic health needs of the people, the Chinese government has been effectively responding to various public health challenges, implementing the policy of “prevention first”, and accelerating the solution of the relatively weak public health, rural and community medical and health work. Especially after the outbreak of the COVID-19 pandemic, the construction of the public health service system has received unprecedented attention, and data show that The National Health Commission issued a total budget target of 58.855 billion yuan for basic public health service subsidy funds in 2022, an increase of 8.473 billion yuan compared with 2020, and the per capita basic public health service subsidy standard increased from 74 yuan in 2020 to 84 yuan in 2022, of which 5 yuan will be added in 2022 to coordinate the epidemic prevention and control work of basic public health services and primary medical and health institutions [2]. After the outbreak of the COVID-19 pandemic, with the strong investment of national public health funds and the continuous revision and adjustment of policies, China has been able to effectively control the continuous epidemic of the COVID-19 pandemic, and the level of national basic public health services has been improved.

In combating the pandemic of Covid-19, however, China’s public health system has fallen in short in a few aspects. For example, the public health resources were found to be allocated in a prominent way of spatial imbalance across and even within the provinces in China. More specifically, the high-quality resources highly converged in relatively developed large cities, which hardly find a way to deliver their quality service along the relatively underdeveloped small cities or towns, let alone the remote rural areas in a province [3]. The spatial imbalance in the provision of public health services affects the effective allocation of medical and health resources among cities. It deviates from the vision of “Healthy China 2030”, which aims for universal health, and contradicts the principles of social fairness and justice. With the development of the concept of universal health and social equity theory, the balance and fairness of medical and health resource allocation have gradually attracted the attention of scholars at home and abroad.

Internationally, Townsend [4] was the first to confirm the existence of imbalance and inequity in the allocation of medical and health services. Subsequent scholars have conducted their own research. For instance, Smith’s [5] study found imbalances in the types of health services provided in wealthy and poor regions: governments tend to provide chronic disease screenings and family doctor services in poorer areas, while providing more rehabilitation and mental health services in wealthier areas. Other scholars [6] have used traditional nearest distance measurement methods to propose a more comprehensive medical and health service accessibility index, through which they evaluated the fairness of Costa Rican residents’ access to healthcare, as well as the impact of health department reforms on healthcare services. In general, after nearly half a century of development, significant progress has been made in foreign studies on the fairness of medical and health services. These studies cover most developed countries and developing countries [7–10], encompassing various levels such as national, city, and community [11–13]. They include various aspects such as the “Balance of medical resource allocation” [14, 15], fairness of healthcare service supply [16, 17], and accessibility of medical service facilities [18, 19].

In China, up to now, most of the previous research has been done to investigate the spatial healthcare allocations across the provinces in China based on inter-provincial panel data [20], but the research remains slim based on intra-provincial panel data, studies on public health care resources in economically developed provinces are even more rare [21]. By the end of 2021, the top 10 provinces with the highest GDP in China are: Guangdong, Jiangsu, Shandong, Zhejiang, Henan, Sichuan, Hubei, Fujian, Hunan and Anhui, most of which are located along the eastern coast of Chinese mainland. Take Guangdong as an example, the province ranks on the top among the provinces in China in terms of gross domestic product (GDP) for decades, its natural population growth rate reaches about 7% and its urbanization rate has reached 71.45% since 2020 [22]. However, the province bears the issue of unbalanced development across cities within its governance [23] and its allocation of healthcare resources is moderately unequal as well [24]. Wei ‘s study also confirmed that although the medical human resources in Guangdong province are consistent with the national level, there are still large regional differences in the allocation of other health resources [25]; Other studies have shown that Guangdong shows a trend of bipolarization between the regions and cities sitting on the Pearl River Delta and the other three regions in its governance, et al., eastern, western and northern Guangdong from the perspectives of economy, education, population and healthcare policies [26]; For example, from 2017 to 2020, the allocation and utilization of health resources in Guangdong province are still concentrated in the Pearl River Delta and other economically developed areas [27]. However, the previous studies have revealed little information about the temporal and spatial distributions and dynamic evolution of public health service supply across its prefectural-level cities. Therefore, it is important to look into the differences in the temporal and spatial allocations of public healthcare resources at a prefecture-level, providing referable information for the government to realize the equality of public health service .

Furthermore, in the study of evaluation methods, various approaches have been employed to measure the supply capacity of health services. For instance, the Analytic Hierarchy Process (AHP) [28], Entropy Weight Method [29], and Cluster Analysis [30] are commonly used evaluation methods. Yang assessed the fairness of basic public services from the output perspective, which did not reflect input-based indicators [31]. Sun et al. constructed an evaluation index system for the equality of medical and health services in rural areas from the perspectives of input, output, and results. However, the result indicators only reflected disease prevention and control, women’s health care, and the proportion of village health rooms [32]. In summary, compared to the method of constructing a system with a single indicator, the academic community prefers to use composite indicators to evaluate the level of health service supply. This is because comprehensive multi-dimensional indicator evaluation methods are relatively more scientific and reliable [33].

Although the existing research has achieved remarkable results, there are still some issues to address: (1) The established public health service evaluation system is relatively simple, even with reasonably-designed output indicators. For example, the inclusion of such attributive indicators as “disease prevention and control” and “maternal and child health level” as part of the output may reduce the accuracy of measurement results [34]; (2) The existing literature mainly focuses on interprovincial panel data, neglecting the fallouts of regional imbalance of healthcare service across intra-provincial areas [3, 35]; (3) The vast majority of studies only compares and analyzes the degree of absolute regional differences in China’s health service, but they have not revealed the relative regional differences, let alone accurate pinpointing of the causes and the composition of their differences, in spite of its relative simplicity and intuition [36]. Based on this, we employ a series of quantitative methods to optimize output and result indicators, exploring the spatial layout, spatiotemporal evolution patterns, and reasons for differences in the level of public health service supply within economically developed provinces using Guangdong Province as a typical case study at the prefecture-level city scale. Further, we distill the mechanisms influencing regional disparities. This not only helps decision-makers more clearly understand the levels of public health services within developed areas, but also provides practical references for government to formulate resource allocation policies in a targeted manner.

**Fig. 1:**
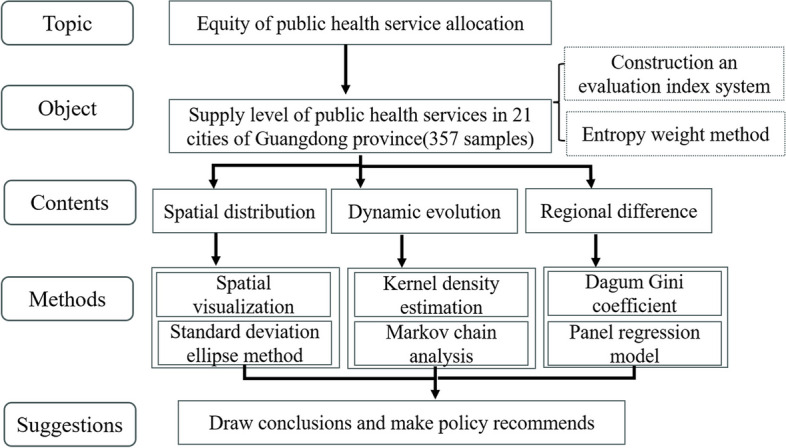
The research framework of this paper

The remainder of this study is organized as follows: Sect. 2 is Materials and Methods. Section 3 ~ 5 are empirical analysis. Section 6 is a discussion. Section 7 sets out conclusions and recommendations. Section 8 concludes the study. Figure 1 shows the framework of this paper.

## Materials and methods

### Guangdong public health service supply level evaluation index system (PHS)

The National Standards for Basic Public Health Services (Third Edition) clearly stipulates 12 basic public health services, including disease prevention and control, maternal and child health care, health education, and health management. According to the connotation of basic public health services in the Code, we follow the principles of systematic, objective, operable and comparable, and expand and optimize the existing indicators based on the views of previous research [20, 37]. On the one hand, “number of medical institutions” and “number of beds” are included in the input index system as material input indicators, and indicators such as “outpatient and inpatient services”, “hospital workload” and “health education” are selected as output indicators. On the other hand, we should expand the outcome indicators such as “maternal and child health care level” and “residents’ health level”, and establish a more comprehensive and reasonable “public health service supply capacity evaluation index system”, as shown in Table 1. The construction of this indicator system takes into account the following factors: Firstly, appropriate inputs and outputs are crucial for meaningful analysis [38], and the combination of input indicators such as resource inputs and output indicators such as treatment numbers can obtain the supply efficiency of medical institutions [39], efficiency measures whether health care resources are used to obtain the best value for money [40], and health care supply efficiency relates to the final health outcomes (outcome indicators) [39]. Among them, the investment in public health services reflects the government’s investment in various resources of medical institutions, including not only financial investment, but also material and human resources. Output indicators should reflect the state of public health service supply, including hospital work efficiency and service efficiency. Among them, Giambrone’s research suggests that the average length of hospital stay is one indicator of hospital work efficiency and medical resource utilization, which can reflect the treatment efficiency and turnover rate of hospital beds [41]. An extended average length of stay may indicate longer treatment periods for patients, leading to slow bed turnover, or it may imply a shortage of hospital bed resources. Therefore, based on data availability, we select the average length of hospital stay as an indicator of hospital work efficiency. Service efficiency is presented from two aspects: outpatient and inpatient services, as well as health education status. Outcome indicators should focus on the basic goals that the government needs to achieve in providing public health services, and reflect the main achievements of public health service provision, such as the level of maternal and child health care, disease prevention and control, and residents’ health [36]. In China, disease prevention and control is one of the important components of basic public health service projects. The incidence and mortality rates of infectious diseases reflect the effectiveness of a region’s public health service in epidemic prevention. Following Chen’s [29]practice, we choose the incidence and mortality rates of Class A and B infectious diseases to measure a region’s level of disease prevention and control. In summary, the evaluation index system of public health service supply selected by us involves multi-dimensional comprehensive consideration of the input, output and outcome of public health services. (2) The evaluation index system should be objective and quantifiable, and we use the mainstream evaluation index system of comprehensive evaluation to evaluate the public health service supply level in Guangdong Province, which can avoid the subjective feelings of groups or individuals and affect the objective judgment of the public health service supply level. (3) Equalization of public health services means that every member of society can receive the same basic public health services, so most of our indicators use per capita indicators rather than general indicators, and a few uses general indicators because such aggregate indicators are more detailed in the health data of prefecture-level cities (such as hospital work efficiency).

**Table 1:** PHS level evaluation index system

First indicators	Secondary indicators	Tertiary indicators	Weight	Quality
Input	Financial investment	Per capita medical and health expenditure/yuan	0.0128	+
		Health expenditure as a proportion of fiscal expenditure/%	0.0275	+
	Material investment	Number of medical institutions per 1,000 people	0.0290	+
		Number of beds per 1,000 population	0.0586	+
	Human input	Number of practicing or assistant physicians per 1,000 people/person	0.0416	+
		Health technicians per 1,000 population/person	0.0783	+
		Number of registered nurses per 1,000 people/person	0.0121	+
Output	hospital work efficiency	Average hospital stay/day	0.0056	-
	Outpatient and inpatient services	Bed occupancy/%	0.0778	+
		The hospital is responsible for the number of consultations/10,000 people	0.1331	+
	Health education	Health check-ups per 10,000 people/10,000 people	0.0685	+
Outcome	The level of disease prevention and control	Incidence rate of Class A and B infectious diseases/1 in 100,000	0.0350	-
		Mortality rate of Class A and B infectious diseases/1 in 100,000	0.0421	-
	Level of maternal health care	Maternal mortality rate/1 in 100,000	0.0428	-
		Maternal hospital delivery rate/%	0.0562	+
		Maternal system management rate/%	0.0452	+
	Level of early childhood care	Infant mortality/‰	0.0864	-
		Health care management rate for children under 7 years of age/%	0.0948	+
	The level of health of the population	Life expectancy per year	0.0520	+

Entropy weight method is an objective weighting method, which determines the objective weight according to the size of the index variability, if the information entropy of an indicator is smaller, indicating the greater the degree of variation in the index value, the more information provided, the greater the role it can play in the comprehensive evaluation, and the greater its weight [42]. Its calculation steps are as follows:

First, in order to make indicators of different dimensions comparable, this paper standardizes positive indicators (Eq. 1) and negative indicators (Eq. 2):

1 $$X_{ij}^{'}=\frac{X_j-X_{min}}{X_{max}-X_{min}},\;1\leq\mathrm i\leq\mathrm m,\;1\leq\mathrm j\leq\mathrm n\;\left(\mathrm{positive}\;\mathrm{indicator}\right)$$

2 $$X_{ij}^{'}=\frac{X_{max}-X_j}{X_{max}-X_{min}},1\leq\mathrm i\leq\mathrm m,\;1\leq\mathrm j\leq\mathrm n\;\left(\mathrm{negative}\;\mathrm{indicator}\right)$$

Second, define the proportion of indicator j in city i.

3 $$P_{ij}=\frac{X_{ij}^{'}}{\sum_{i=1}^mX_{ij}^{'}},\;\left(0\leq{\mathrm P}_{\mathrm{ij}}\leq1\right)$$

Third, find the entropy value е_j_ of the value index j to obtain the information entropy redundancy d_j_:

4 $${e}_{j}=-\frac{1}{lnm}\sum _{i=1}^{m}{P}_{ij}{lnP}_{ij}$$

5 $${d}_{j}=1-{e}_{j}$$

Fourth, (4) and (5) can confirm that the weight of indicator j is W_j_:

6 $${W}_{j}=\frac{{d}_{j}}{\sum _{i=1}^{m}{d}_{i}}$$

Finally, the formula for calculating the PHS level in Guangdong Province is obtained:

7 $$PHS=\sum_{i=1}^mW_jy_{ij},\;0\leq PHS\leq1$$

### Spatial distribution analysis methods

The standard deviation ellipse method is one of the classic methods to analyze the directionality characteristics of spatial distribution, the size of the ellipse reflects the concentration of the overall elements of the spatial pattern, and the declination angle (major semi-axis) reflects the dominant direction of the pattern [43]. Method can calculate the center of gravity distribution, major axis standard deviation, minor axis standard deviation, azimuth angle and other parameters [29]. We used the standard deviation ellipse method to study the spatial distribution characteristics of public health service supply level in Guangdong Province. The specific steps are as follows:

8 $$\stackrel{-}{{X}_{w}}=\frac{\sum _{i=1}^{n}{w}_{i}{x}_{i}}{\sum _{i=1}^{n}{w}_{i}}\stackrel{-}{{Y}_{w}}=\frac{\sum _{i=1}^{n}{w}_{i}{y}_{i}}{\sum _{i=1}^{n}{w}_{i}}$$

$${\sigma }_{x}=\sqrt{\frac{\sum _{i=1}^{n}{({w}_{i}\stackrel{-}{{x}_{i}}\text{cos}\theta -{w}_{i}\stackrel{-}{{y}_{i}}\text{sin}\theta )}^{2}}{\sum _{i=1}^{n}{{w}_{i}}^{2}}}$$

9 $${\sigma }_{y}=\sqrt{\frac{\sum _{i=1}^{n}{({w}_{i}\stackrel{-}{{x}_{i}}\text{sin}\theta -{w}_{i}\stackrel{-}{{y}_{i}}\text{cos}\theta )}^{2}}{\sum _{i=1}^{n}{{w}_{i}}^{2}}}$$

10 $$\text{tan}\theta=\frac{\left(\sum_{i=1}^nw_i^2\overset-{x_i^2}-\sum_{i=1}^nw_i^2\overset-{y_i^2}\right)+\sqrt{{(\sum_{i=1}^nw_i^2\overset-{x_i^2}-\sum_{i=1}^nw_i^2\overset-{y_i^2})}^2+4\sum_{i=1}^nw_i^2\overset-{x_i^2}\overset-{y_i^2}}}{2\sum_{i=1}^nw_i^2\overset-{x_i}\overset-{y_i}}$$

where W_i_ stands for weight; θ represents the azimuth of the standard deviation ellipse, which is the clockwise angle formed by the long axis of the standard deviation ellipse;, σ_x_ and σ_y_ represent the standard deviation on the x axis and y axis, respectively.

### Dynamic evolution analysis method

#### Kernel density estimation method

The kernel density estimation method of nonparametric estimation can use the observed sample values to estimate the probability density of the data without using prior knowledge and unknown event probability distribution, and make full use of the sample data to better reflect the distribution position, morphology, ductility and polarization characteristics of variables, which is common in spatial non-equilibrium analysis. Therefore, our study uses this method to estimate the distribution of PHS levels in Guangdong Province. According to the research of Ge [44], by comparing the distribution curves of different periods, the dynamic characteristics of PHS level can be analyzed, the change trend of the overall position reflects the level of PHS level, the change of the height and width of the main peak reflects the change trend of the absolute difference in supply capacity between prefecture-level cities, the ductility of the distribution form can examine the gap between prefecture-level cities with high supply capacity and prefecture-level cities with low supply capacity, and the number of peaks can explain the polarization degree of supply capacity. The basic principle is as follows:

11 $$F\left(x\right)=\frac{1}{nh}\sum _{i=1}^{n}K\left(\frac{{X}_{i}-\alpha }{h}\right)$$

In Eq. (11), α represents the average; X_i_ represents the observation of independent homogeneous distribution; h represents bandwidth; K(*) represents the kernel function.

#### Markov chain analysis method

Since the kernel density estimation method cannot reflect the relative position change between the level of public health services in the region and the possibility of change, we introduce the traditional Markov chain analysis method to characterize the dynamic evolution of public health service supply level in Guangdong Province. Referring to the practice of Chen [36], we synthesized and scattered the PHS level in Guangdong Province into k types during the observation period, and constructed the state transition probability matrix E of *K×K* to measure the transfer of the comprehensive score between different types in different periods. In this paper, the probability distribution of the t-year PHS horizontal state is expressed as the state probability vector E_t_ of 1×*K* ( Eq. 12). In Eq. (13), E_pq_ represents the probability of a place moving from the p type in the t period to the q type in the t + 1 period; k_pq_ represents the number of times the transfer from the p type in the t period to the q type in the t + 1 period occurs; k_i_ indicates the total number of occurrences belonging to type i during the observation period.

12 $$E=\left[{E}_{1,t},{E}_{2,t},\dots ,{E}_{k,t}\right]$$

13 $${E}_{pq}=\frac{{k}_{pq}}{{k}_{p}}$$

However, the traditional Markov chain treats the 21 prefecture-level cities in Guangdong Province as independent regions without considering spatial spillover effects. In fact, with the in-depth development of socio-economic and medical system reform, the relative independence of medical and health services between different regions has been gradually weakened, and exchanges and cooperation between regions have become increasingly close, and the development of local medical and health services is bound to be affected by the development of medical and health services in neighboring cities. Therefore, we refer to related research [45] to introduce the concept of “spatial lag” in the traditional Markov chain model to investigate the influence of geospatial factors on the probability of local public health service supply capacity shift, and reveal the relationship between the temporal and spatial evolution trend of observation objects and geospatial factors. According to the spatial lag type of the initial time of a certain region, the traditional Markov chain transfer probability matrix *K×K* is decomposed into K *K×K* transfer probability matrix, and the spatial lag type of a prefecture-level city in t year is K, then, E_pq_ (K) represents the probability of spatial transfer to state q in t + 1 year conditional on the spatial lag type K of the prefecture-level city in t year. Assuming that the spatial lag level of the region is determined by its spatial lag value, the spatial lag value is the weighted average of the observation values of the adjacent areas around a certain area, and the state of the adjacent regions is judged by introducing a spatial weight matrix. The calculation formula is as follows:

14 $$Lag=\sum _{q}{Y}_{q}{W}_{ij}$$

In Eq. (14), Lag is the spatial lag value; Y_q_ represents observations in adjacent areas; and W_ij_ represents the spatial weights matrix.

### Regional difference analysis method

#### Dagum gini coefficient and decomposition method

We used the Gini coefficient proposed by Dagum [46] and its decomposition method to analyze the regional differences in the level of public health service supply in Guangdong Province. Specifically, the overall Gini coefficient G can be decomposed into three parts [29]: intra-group difference contribution G_w_, between-group difference contribution G_nb_ and supervariable density contribution G_t_, representing the effects of intra-group differences, between-group differences and inter-group cross-overlap differences on the overall difference, respectively, G = G_nb_+G_w_+G_t_. The calculation formula is shown in Eq. (15):

15 $$G=\frac{\sum _{j=1}^{\tau }\sum _{h=1}^{\tau }\sum _{i=1}^{{n}_{j}}\sum _{r=1}^{{n}_{h}}\left|{X}_{ji}-{X}_{hr}\right|}{2\gamma {n}^{2}}$$

In Eq. (15), n represents the total number of samples during the survey period; τ indicates the number of prefecture-level cities; N_j_ and N_h_ indicate the number of prefecture-level cities in the area where j and h are located, respectively; γ indicates the average level of the region’s public health service supply capacity; X_ji_ and X_hr_ respectively indicate the public health service supply level of the prefecture-level cities where j and h are located.

#### Panel regression model

In order to further analyze the main factors affecting the spatial difference of public health service supply in Guangdong Province, we take the public health service supply level index (PHS) as the dependent variable and the regional economic development level, regional fiscal freedom, population factors, urbanization level and industrial structure upgrading as the independent variables to test the influencing factors of the spatial difference of public health service supply in Guangdong Province. Regression model settings can be found in Eq. (16):

16 $${PHS}_{it}={\alpha }_{i}+{\delta }_{n}\sum _{n-1}^{n}{X}_{itn}+{\epsilon }_{it}$$

where i represents a prefecture-level city (i = 1,2, …,N); t represents time (t = 1,2, …,T);, X_it_ represents the core arguments; n is the number of independent variables (*n* = 1,2, …,N);, α_i_ are cross-sectional terms; ε are random perturbation terms.

#### Threshold effect model

Based on the results of the influencing factors in this study, we draw on the research approach of Hansen (1999) [47] to further verify whether there is a nonlinear effect of the factors that have a significant influence on the supply of public health services, in order to explore the underlying mechanism of action. Some studies have found that [48]: Both urbanization rate and fiscal capacity are based on the level of regional economic development;The higher the level of regional economic development, the higher the level of urbanization, the stronger its fiscal extraction capacity, the greater its fiscal capacity, and the more conducive it is to the supply of public health services by local governments. This suggests that the level of regional economic development has a significant indirect effect on the supply of public health services, with different levels of economic development potentially corresponding to different degrees of public health service supply capacity. Therefore, this paper selects the level of regional economic development as the threshold variable, and uses the threshold effect model to examine the indirect effects of urbanization rate and fiscal decentralization on the supply of public health services in different threshold intervals. The basic formula for the threshold effect is (17):

17 $$y_{it}=\mu_i+\beta_1x_{it}\cdot I\left(q_{it}\leq\gamma\right)+\beta_2x_{it}\cdot I\left(q_{it}>\gamma\right)+\epsilon_{it}$$

In the formula, i represents the region, t denotes the year, ${q}_{it}$ signifies the threshold variable, $\gamma$ represents the estimated threshold value, ${\epsilon }_{it}$ is the random disturbance term, and I(·) stands for the indicator function.

#### Core arguments

Indeed, some articles specifically address the social, economic and environmental drivers of equity in the allocation of health resources, but imbalances in the allocation of health resources in most regions are determined by external factors in the health sector [49, 50]. We found that the main factors affecting the level of health service provision are as follows: (1) We first consider the level of economic development (PGDP), generally speaking, affected by the size of the regional population, we will use per capita GDP to measure the overall level of economic development of a region. The level of regional economic development is the financial basis for ensuring the local supply of sufficient basic public health services, and the equalization of financial resources is a necessary condition for promoting the equalization of basic public health services. (2) Regional fiscal freedom (FSS): measured by the ratio of local budget revenue to general budget expenditure. In China, the magnification of fiscal power has strengthened the role of the government in providing health services [51]; Generally speaking, the greater the degree of regional fiscal decentralization and the more free the local government is in the grasp of finance, the more power rent-seeking is likely to occur, thereby expanding the input of local public health service supply, which can promote the improvement of public health service supply capacity and accelerate the pace of equal allocation [29]. (3) Population factor (DEN), we express it in terms of population density. Studies have shown that under the constraints of government resources, the greater the population density of the region, the more people share public health services, and the per capita level will decline, which makes it more difficult to equalize basic public health services [52]. (4) The urbanization level (UR) is reflected by the proportion of urban population at the prefecture-level city and the permanent population at the end of the year, which reflects all aspects of regional social economy, and the promotion of urbanization rate is conducive to the improvement of public health service supply level [52]. (5) Industrial Structure Upgrading (ISU), we measure by the ratio of the added value of the tertiary industry to the regional GDP. With the advent of the digital age, the traditional social and economic structure is also being optimized and upgraded, and digital technology has promoted the popularization of Internet medical care at the people’s level, greatly facilitating people’s lives, enriching residents’ consumption patterns and consumption choices, and giving birth to rich and diversified health service needs, which further urge local governments to accelerate the improvement of public health service supply level [53, 54]. To eliminate dimensional differences, we treat the control variable non-ratio data logarithmic ally. Table 2 shows the descriptive statistics of variables.

**Table 2:** Summary statistics of variables (*N* = 357)

Variables	Mean	Standard deviation	Min	Max
PHS	0.3923	0.0578	0.2608	0.5957
LnPGDP	10.5832	0.7860	8.9211	13.0557
FSS	0.5282	0.2429	0	3.5942
lnDEN	6.3638	0.7079	5.1509	8.2216
UR	0.8332	0.3028	0	3.5942
ISU	43.0377	9.6762	0	76.0700

#### Data sources and regional division

**Fig. 2:**
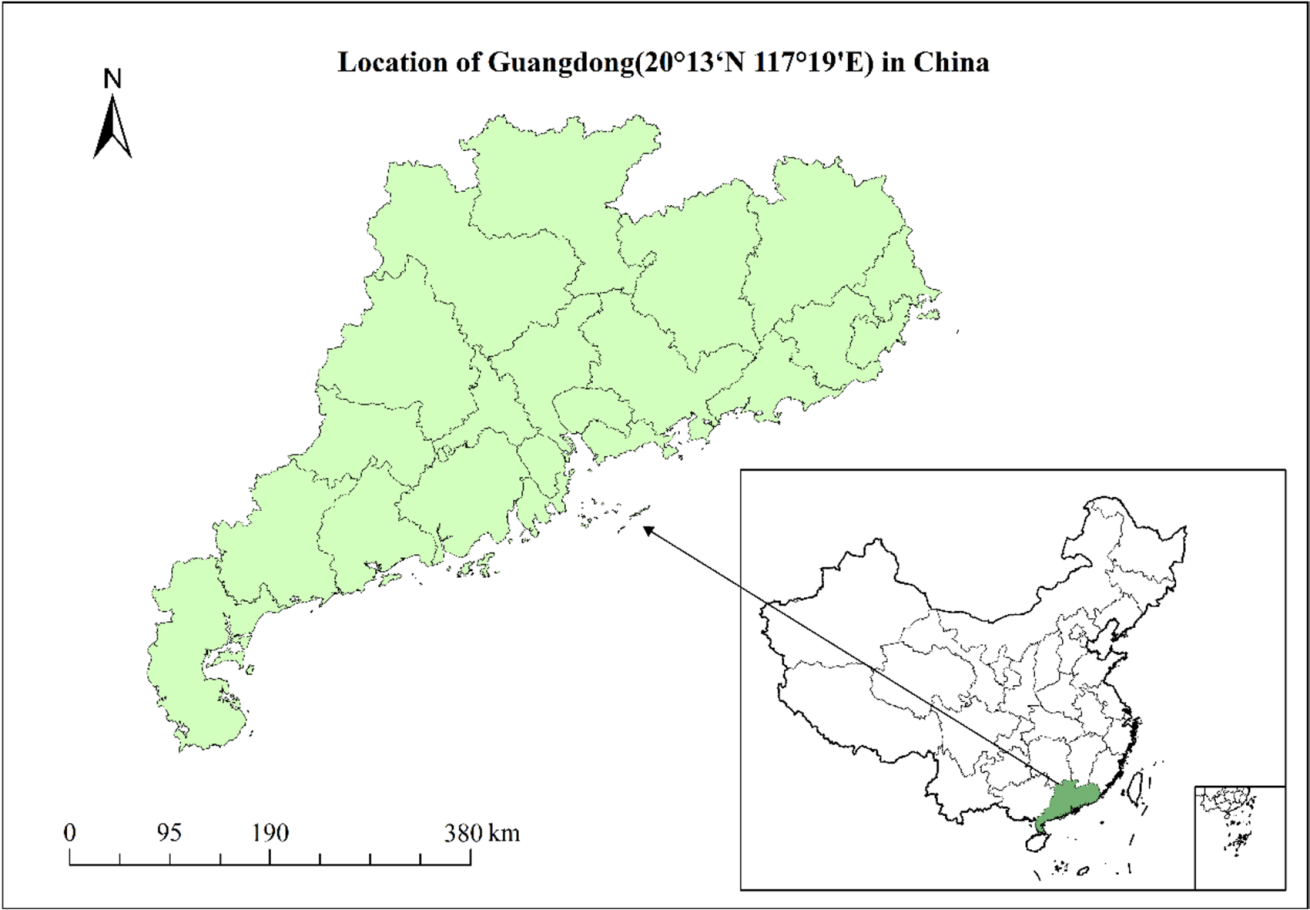
Study area

In this paper, the data of 21 prefecture-level cities in Guangdong Province were used, including a total of 357 research samples from 2005 ~ 2021, and the research area is shown in Fig. 2. The data used are from the Guangdong Provincial Health Statistics Yearbook and the Simplified Health Statistics of Guangdong Province (http://wsjkw.gd.gov.cn/) provided on the portal of the Guangdong Provincial Health Commission; Statistical Bulletin on National Economic and Social Development provided by municipal governments at all levels; and the Statistical Yearbook (http://stats.gd.gov.cn/) provided by Guangdong Statistical Information Network. The administrative map is obtained from the Resource and Environment Data Cloud Platform (http://www.resdc.cn). In addition, we use linear interpolation to handle missing data. According to the division of economic regions of Guangdong Province by the Bureau of Statistics of Guangdong Province in 2019, it can be divided into four major regions: the Pearl River Delta, eastern Guangdong province, western Guangdong province and northern mountainous areas of Guangdong province (Table 3). Overall, there are significant differences in economic development, natural resource status and geographical climate between the four regions. Our analysis of these four regions can examine the fairness of public health service supply in Guangdong Province from a regional perspective.

**Table 3:** Guangdong’s four main regions

Regions	Perfecture-level city
Pearl River Delta	Guangzhou, Shenzhen, Zhuhai, Foshan, Huizhou, Dongguang, Zhongshan, Jiangmen, Zhaoqing
Eastern Guangdong province	Shantou, Shanwei, Chaozhou, Jieyang
Western Guangdong province	Yangjiang, Zhanjiang, Maoming
Northern Guangdong province	Shaoguan, Heyuan, Meizhou, Qingyuan, Yunfu

### Spatial distribution analysis results

#### Factual description

**Fig. 3:**
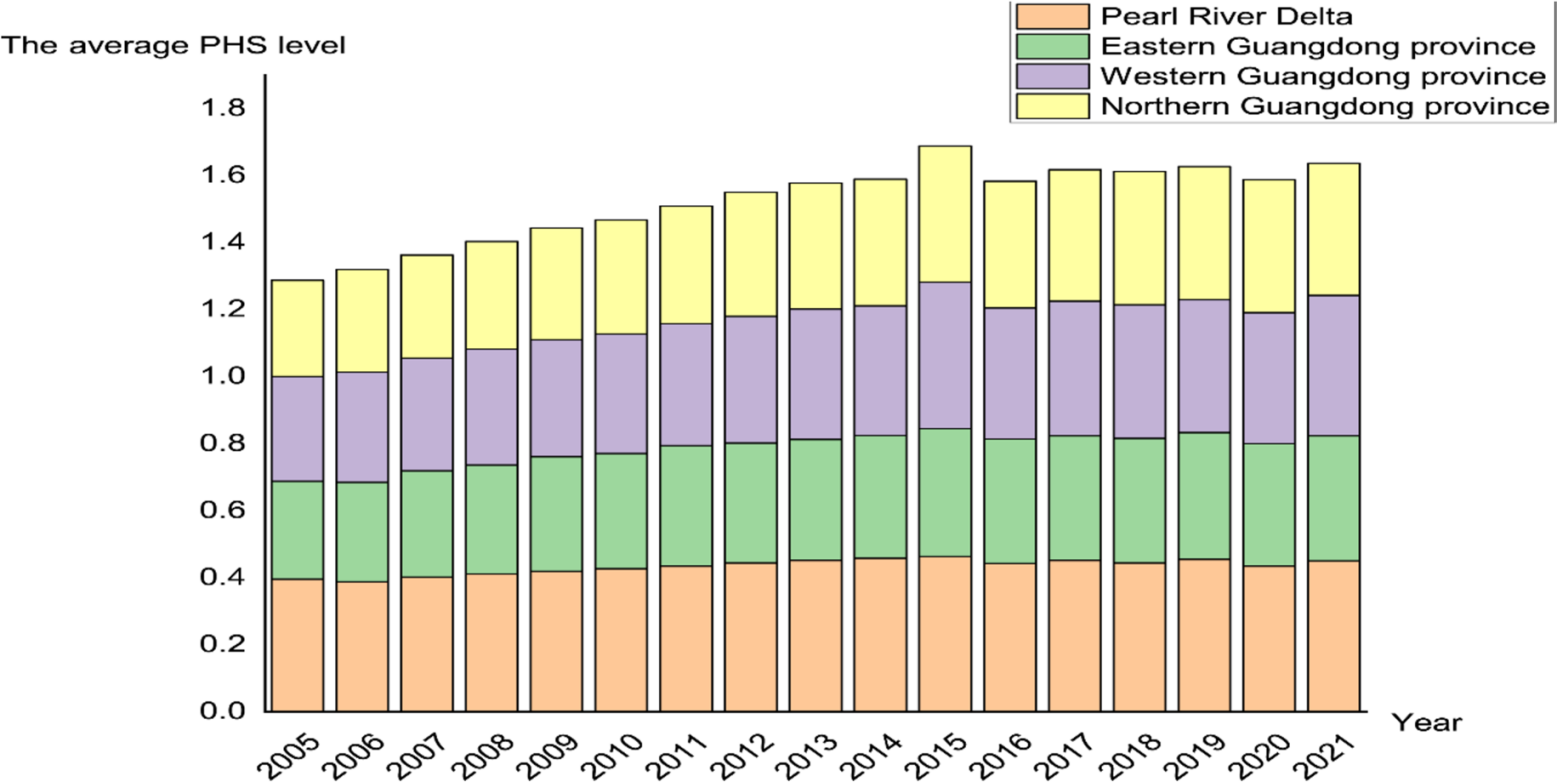
The average PHS level of four regions from 2005 to 2021

Based on the results of the PHS evaluation index system, we calculated the comprehensive scores of 21 prefecture-level cities in Guangdong Province from 2005 ~ 2021, and the results are shown in Table A1 in the appendix. As can be seen from Fig. 3, in general, taking 2015 as the turning point, the PHS supply level showed a stable and continuous rise and was in a stable fluctuation and upward trend, and the PHS level in Guangdong Province scored the highest in 2015. From the perspective of sub-regions, the supply level of PHS in the Pearl River Delta region and eastern Guangdong region has remained relatively stable over the years; It is worth noting that the PHS level in northern Guangdong and western Guangdong showed a continuous upward trend in 2005 ~ 2015, but the PHS level after 2015 fell and was in a stable fluctuation and rising state.

**Fig. 4:**
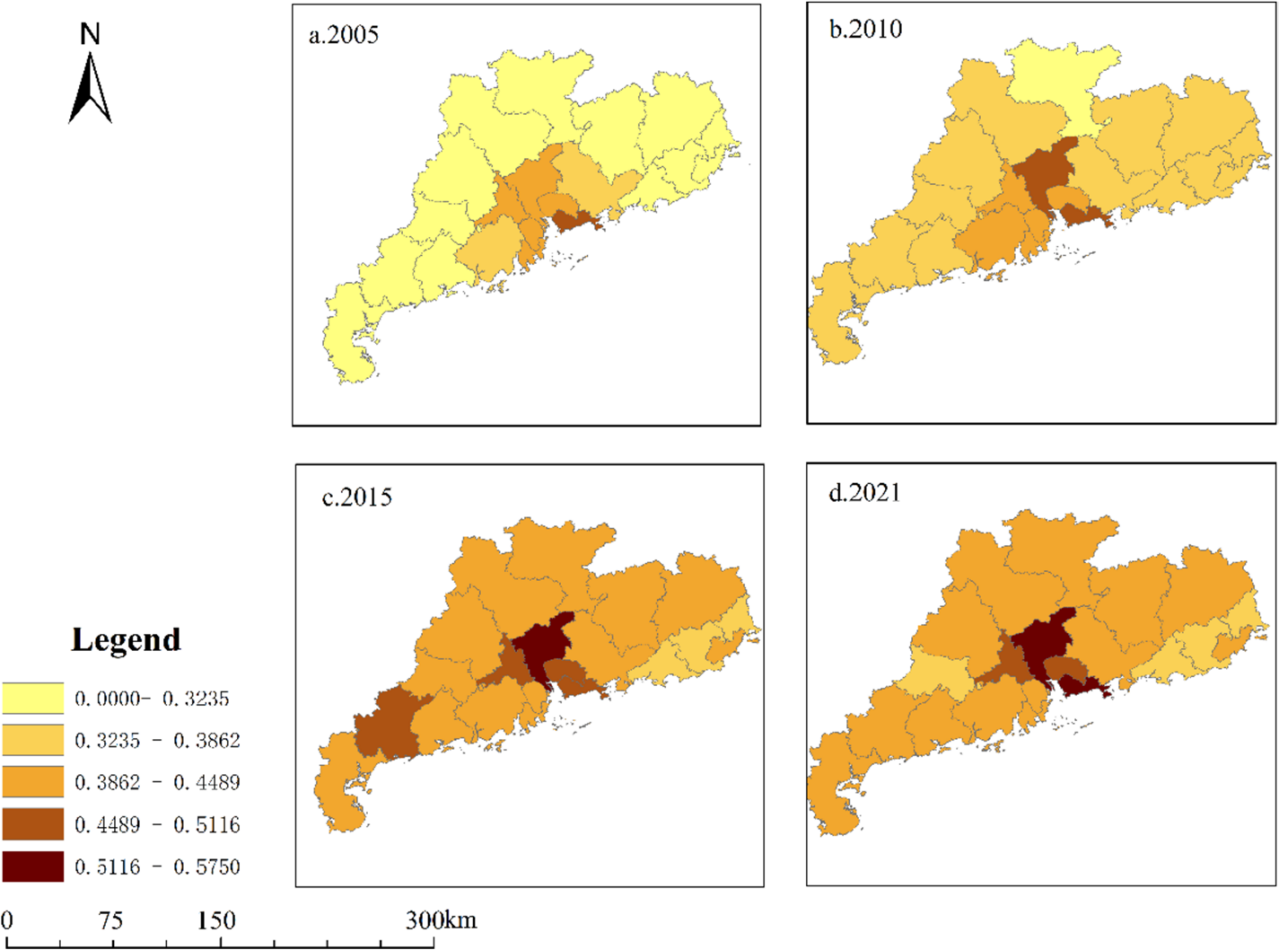
The spatial distribution of the PHS level in Guangdong

In order to analyze the distribution characteristics of PHS levels in various cities in Guangdong Province from the perspective of spatial distribution, we selected the comprehensive scores of public health service supply levels of prefecture-level cities in Guangdong Province in 2005, 2010, 2015 and 2021 as sample data, and used ArcGIS 10.2 software to visualize them by mapping them. In this paper, we use the quintile method to divide the comprehensive score of PHS in the study area into five levels: high (0.5116 ~ 0.5750), high (0.4489 ~ 0.5116), medium (0.3862 ~ 0.4489), low (0.3235 ~ 0.3862) and low (0.0000 ~ 0.3235). From Fig. 4, we can find that the PHS level of various cities shows a relatively obvious growth trend. Specifically, the overall PHS level in Guangdong Province in 2005 was still relatively low, the PHS level in western Guangdong, northern Guangdong and eastern Guangdong is at a low level, while the Pearl River Delta region is at a medium level, and the PHS level gap between marginal cities and developed cities is large. By comparing the spatial visualization maps in 2010, 2015 and 2021, the PHS level in Guangdong Province has improved, and the relative differences between local cities have decreased significantly, but in general, it is still at a high and stable level in the Pearl River Delta region in previous years. In addition, it is worth noting that the PHS level in western Guangdong shows a fluctuating upward trend, for example, by 2021, the PHS level in western Guangdong is at a relatively medium level, which is not much different from 2015; However, from the perspective of prefecture-level cities, the PHS levels of Maoming City and Yunfu City in western Guangdong in 2015 were higher than those in other years.

#### Spatial and temporal change analysis

**Fig. 5:**
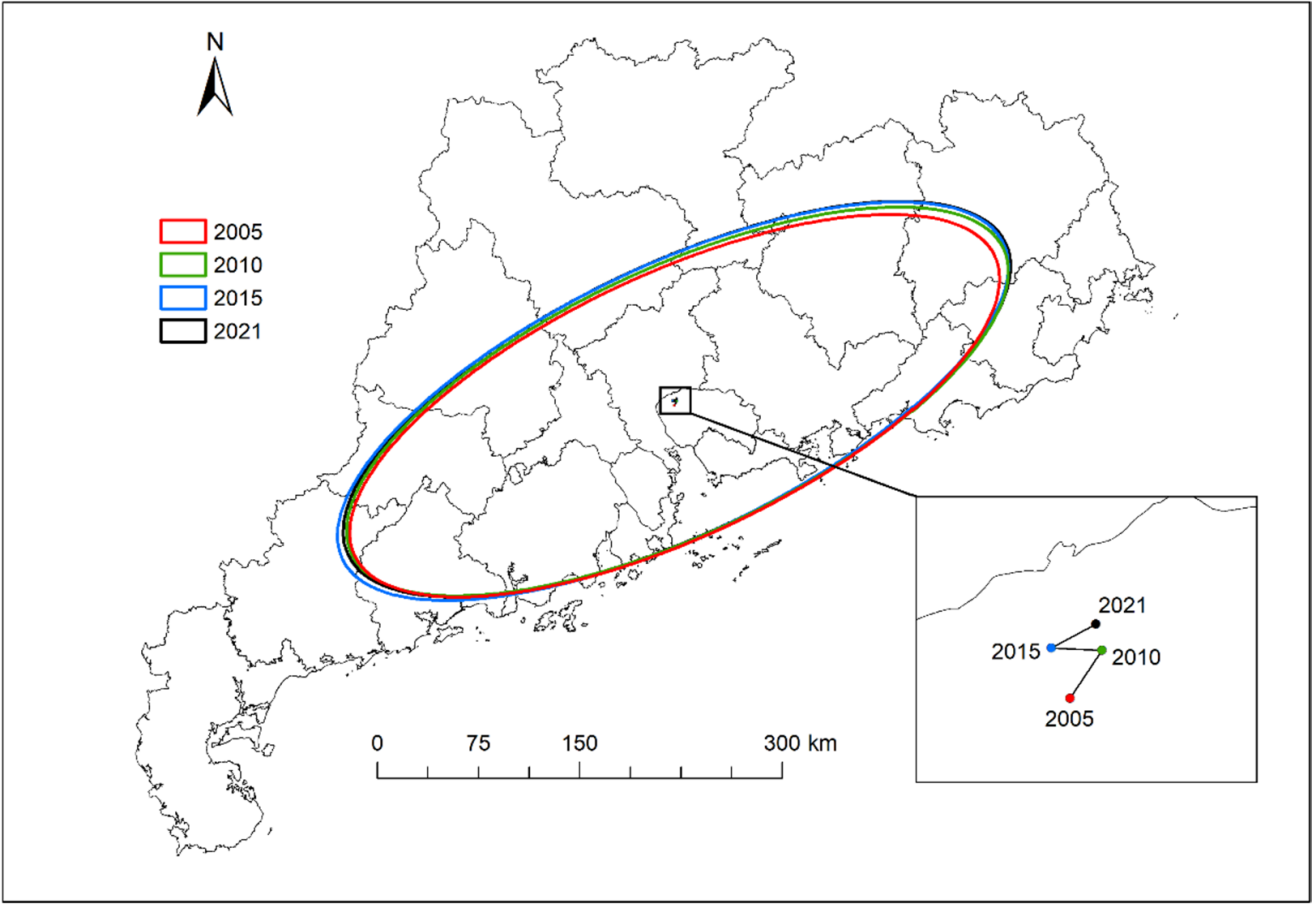
Standard deviation ellipse distribution of PHS level in Guangdong province in different years

The standard deviation ellipse method measures the direction and aggregation of a set of data and is used in the study of healthcare access [55, 56]. In this paper, the standard deviation ellipse method was used to calculate the spatial distribution center of gravity, distribution morphology and distribution direction of public health service supply level in Guangdong Province in four characteristic years in 2005, 2010, 2015 and 2021. It can be seen from Fig. 5 that the horizontal spatial distribution center of PHS in Guangdong Province in 2005 ~ 2021 is located in Dongguan City (114°15’E, 23°09’N) in the Pearl River Delta region, which basically shows the spatial distribution pattern of “southwest-northeast”. Among them, the center of gravity shifted from south to northeast in 2005 ~ 2010, slightly shifted to the west in 2010 ~ 2015, and shifted slightly to the northeast in 2015 ~ 2021, which shows that the center of gravity moved to the northeast for the longest time, indicating that the level of the center of gravity in northern Guangdong rose faster than the average level of Guangdong Province. From the perspective of elliptical area, the elliptical area gradually increased in 2005 ~ 2021, indicating that there was a trend of spatial concentration to spatial differentiation in the allocation of public health resources in Guangdong Province. From the perspective of azimuth angle, the change range of azimuth is small, and the change trend is relatively stable, from 64.94° in 2005 to 64.61° in 2021, indicating that the standard deviation ellipse shows clockwise rotation in spatial distribution, and the PHS level in the southwest or northeast regions of the ellipse increases rapidly. From the perspective of the length of the semi-axis, the length of the major semi-axis increased from 97341.23 km in 2005 to 99651.89 km in 2021, and the length of the semi axial increased from 261519.99 km in 2005 to 269247.73 km in 2021, indicating that the spatial agglomeration and differentiation of PHS level in Guangdong Province decreased during the study period.

### Dynamic evolution analysis results

#### Kernel density estimation

**Fig. 6:**
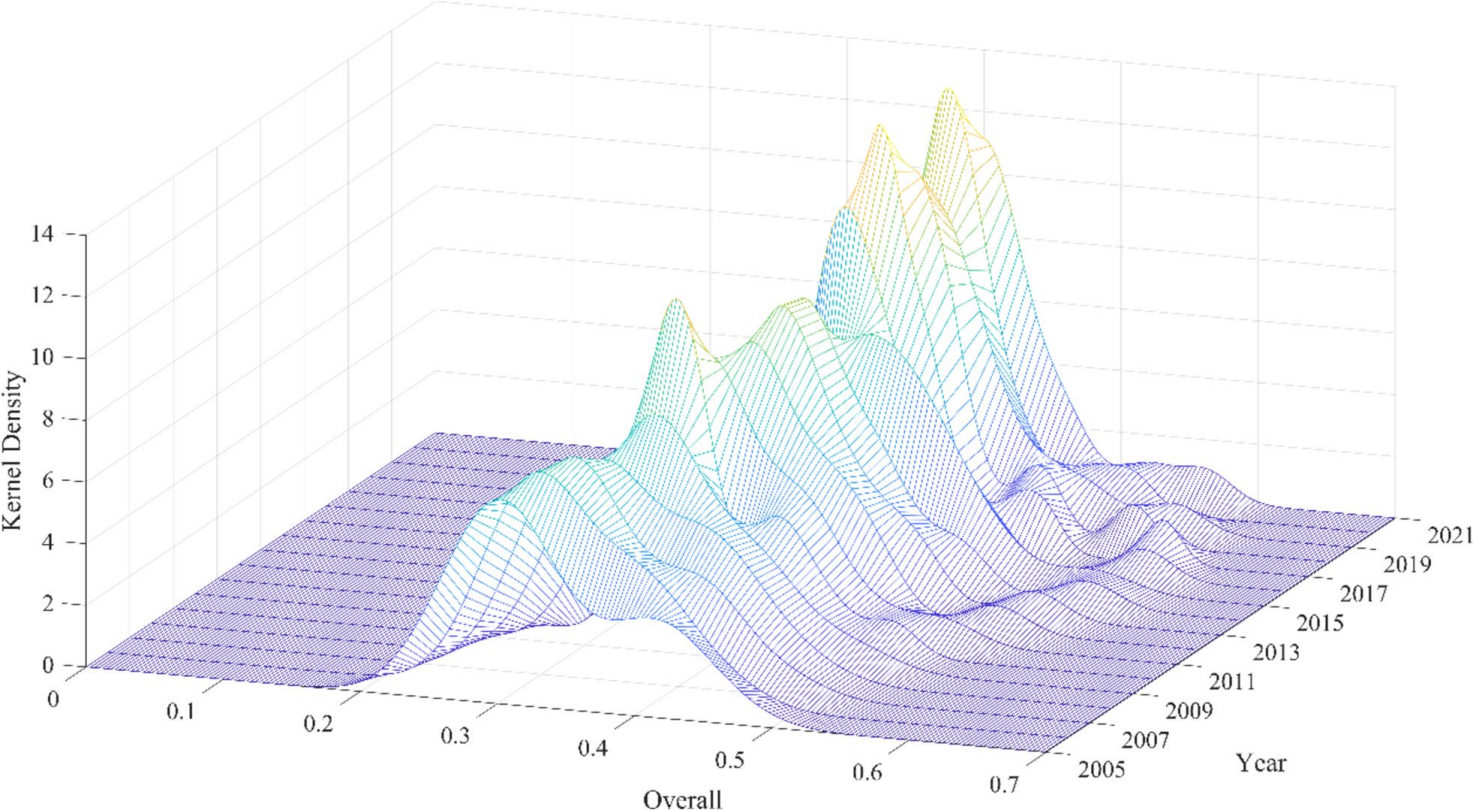
Distribution dynamics of PHS overall level in Guangdong province

**Fig. 7:**
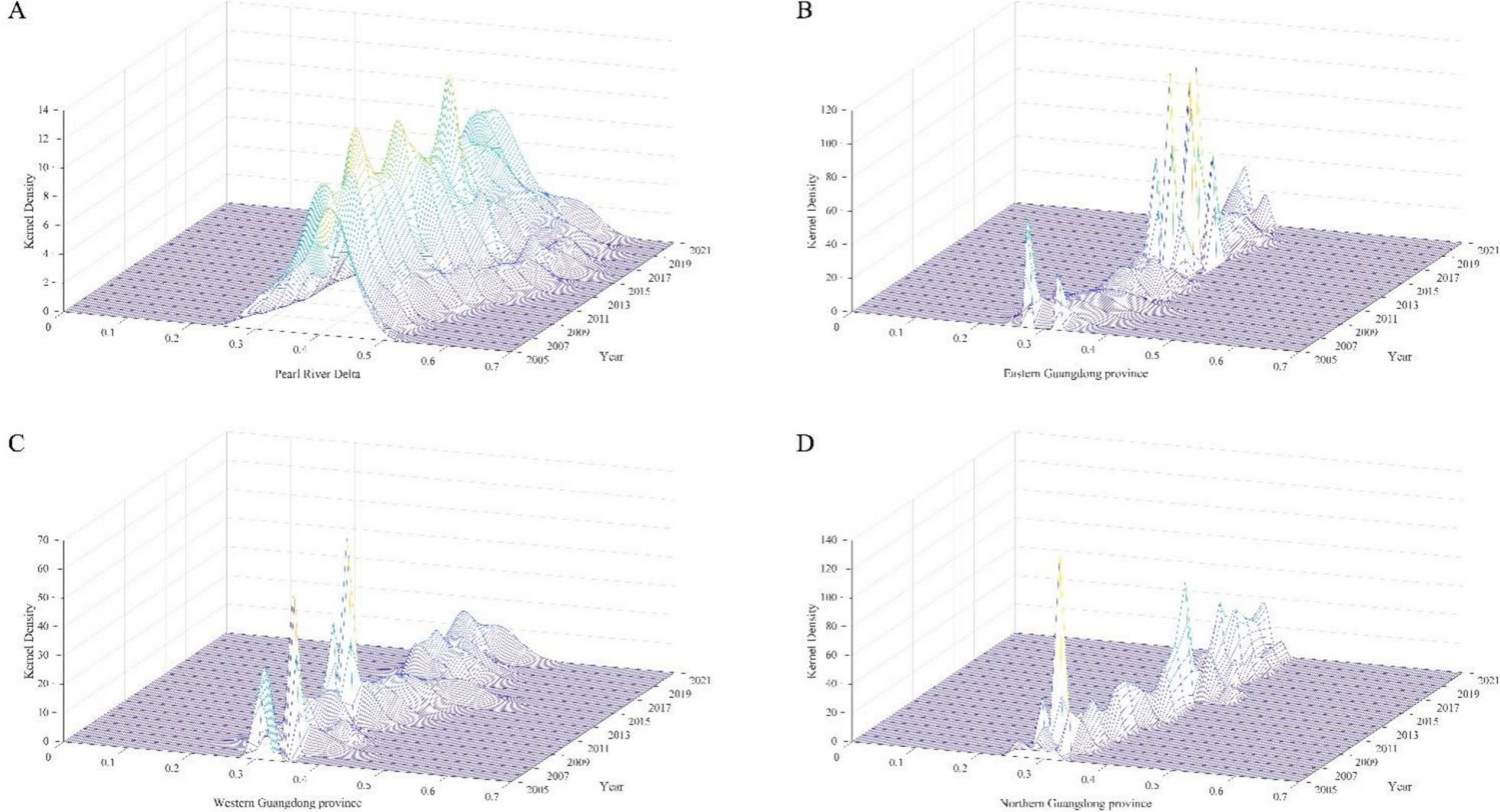
Distribution dynamics of PHS overall level in Guangdong province

In order to obtain dynamic information on the absolute difference in public health service supply level between regions in Guangdong Province, Metlab2021 software was used to estimate the kernel density, and the results are shown in Figs. 6 and 7. Figure 6 shows the overall PHS level in Guangdong Province. Firstly, the center of the kernel density distribution curve at the overall level of PHS in Guangdong Province shifted to the right with the increase of the year, indicating that the public health service supply level of prefecture-level cities in Guangdong Province evolved from low level to high level during the observation period. Secondly, looking at the peak trends of the distribution curve, the main peak height has a slight rise before showing a fluctuating downward and then upward trend. Additionally, the width of the curve exhibits a process of broadening before slightly narrowing. This implies that the absolute differences in the levels of public health service supply across prefecture-level cities in Guangdong Province have generally decreased on the whole. Thirdly, from the perspective of distribution ductility, the distribution curve of Guangdong Province showed a phenomenon of right tailing and continued to expand, indicating that the gap between prefecture-level cities with high public health service supply level (Guangzhou, Shenzhen, Foshan, etc.) and prefecture-level cities with low supply level (Shanwei, Jieyang, Chaozhou) gradually widened during the observation period. Fourthly, from the perspective of polarization, the number of peaks at the PHS level is manifested as the change process of “one main side bimodal - inconspicuous bimodal”. This indicates that the PHS supply capacity in Guangdong Province has a certain gradient effect and has two-level differentiation characteristics, but the differentiation trend gradually weakens during the observation period, and finally presents a weak two-level differentiation phenomenon.

Then, Fig. 7(A) shows the dynamic distribution of public health service supply levels in the Pearl River Delta region of Guangdong Province. First, from the distribution position, the center point showed a trend of “moving to the right to the flat”, indicating that the PHS level in the Pearl River Delta region gradually rose to flatten. Second, from the perspective of distribution pattern, the height of the main peak of the distribution curve decreased slowly, indicating that the PHS level in the Pearl River Delta region showed a trend of slow divergence. Third, from the perspective of distribution ductility, the distribution curve shows the phenomenon of “left drag to right drag change”, “left drag” first narrows and then “right drag” changes, and “right drag” first shrinks and then widens. Finally, from the perspective of polarization, the distribution curve of the Pearl River Delta region is in the distribution trend of “single peak - one main side double-peak”, indicating that there is polarization in the Pearl River Delta region.

In addition, the dynamic evolution trend of the distribution of public health service supply levels in eastern Guangdong can be seen from Fig. 7(B). First, the center point of the curve tends to move to the right, indicating that the pH level in this area is gradually increasing. Second, the main peak height of the distribution rises slightly. Third, the curve in eastern Guangdong is the distribution state of “weak bimodal-unimodal - weak bimodal”, indicating that there is weak polarization in this region.

Further, Fig. 7(C) shows the dynamic evolution trend of the distribution of public health service supply levels in western Guangdong. First, PHS levels are gradually increasing in the region, consistent with other regions. Second, the main peak height of the distribution fluctuated greatly, the overall height decreased slightly, and the curve width showed a change process of “greatly widening-gradually narrowing-gradually widening”, indicating that the absolute difference in PHS levels in western Guangdong expanded with time. Third, most of the curves in western Guangdong are “unimodal distribution”, indicating that there is no polarization in this region.

Finally, Fig. 7(D) shows the dynamic evolution trend of the distribution of public health service supply levels in northern Guangdong. First, PHS levels are gradually increasing in the region, consistent with other regions. Second, the height fluctuation range of the main peak is small, the height of the main peak is significantly higher than that of other regions, and the width of the main peak is also significantly narrower than that of the other three regions, indicating that the absolute difference in PHS level in northern Guangdong is smaller than that in other regions. Thirdly, the initial curve for the northern Guangdong region exhibits a “bimodal” distribution state. With the increase in years, it presents a “unimodal-weakly bimodal” condition, indicating a weak polarization phenomenon in this region.

### Markov chain analysis

#### Analysis of dynamic characteristics of traditional markov chains

The kernel density estimation method can only simply describe the temporal trend of PHS level in Guangdong Province, but cannot analyze its intrinsic distribution dynamic trend and characteristics, and the advantage of the Markov chain method is that it provides dynamic information about the movement of the region within the distribution [45]. Therefore, our paper also adopts the traditional Markov chain analysis method to divide the PHS level of 21 prefecture-level cities into four different types of regions according to quartiles in 2005 ~ 2021: I indicates low level (≤ 0.3558), II represents medium and low level (0.3558 ~ 0.3578), III indicates medium and high level (0.3578 ~ 0.4234), IV indicates high level (≥ 0.4234), according to this, the probability matrix of PHS level state transition in Guangdong Province is measured, and the results are shown in Table 4. The elements on the main diagonal indicate the probability that the state type of public health service supply level in Guangdong Province has not shifted, reflecting the stability of PHS level. Elements on non-diagonal lines represent the probability of transitions between different state types. From the results of Table 4, first of all, we can find that the values on the main diagonal are significantly higher than the values on the non-diagonal, Specifically, the probability of maintaining stability for type I, type II, type III and type IV were 78.89%, 62.35%, 58.97% and 81.93%, respectively; The probability of type I transferring to type II is 20.00%; The probability of type II transferring to type III is 30.59%; The probability of type III transferring to type IV is 21.80%. Secondly, the probability of type II to type I, type III to type II, and type IV to type III are: 5.88%, 19.23%, and 15.66%, respectively. In addition, there are also cases of leapfrog transfer but the probability is small, which are 1.11%, 1.18% and 2.41%, respectively, which indicate that the PHS level in Guangdong Province has a club convergence effect during the observation period, and the overall upward improvement is high, and the possibility of leapfrog development is low.

**Table 4:** Traditional markov chain transfer probability matrix of PHS level in Guangdong province

	I	II	III	IV
I	0.7889	0.2000	0.0111	0.0000
II	0.0588	0.6235	0.3059	0.0118
III	0.0000	0.1923	0.5897	0.2180
IV	0.0000	0.0241	0.1566	0.8193

#### Dynamic feature analysis of spatial Markov chain

The traditional Markov chain analysis process treats each region as an independent unit and does not take into account the impact of geospatial factors on the allocation of public health services, but in fact, we need to know that the upward or downward shift of the public health service supply level is not spatially isolated. Therefore, with reference to the research of spatial analysis [45, 57], we incorporate the spatial lag factor into the traditional Markov chain probability transfer matrix, construct the spatial Markov chain probability transfer matrix, and re-analyze the dynamic spatial evolution trend of PHS level in Guangdong Province on the basis of the new matrix. It can be seen from Table 5 that after including the spatial lag factor, (1) The influence of spatial factors on the transfer of PHS level in Guangdong Province was obvious, for example, when Type I was transferred to Type II without considering geospatial factors, the probability was 20.00%; However, after considering the spatial factors, the probabilities were: 15.52%, 19.23%, 60.00% and 100.00%, respectively. (2) The stability of PHS levels in the same type varies significantly depending on the adjacent type. For example, the stability probabilities of type I in adjacent types I, II, III, and IV were: 84.48%, 76.92%, 40.00%, and 0, respectively. The stability probabilities of type II in adjacent types I, II, III and IV were 65.22%, 72.00%, 54.17% and 53.85%, respectively. For type III, the stability probabilities in adjacent types I, II, III and IV were 57.14%, 62.50%, 61.54% and 52.38%, respectively. For type IV, the stability probabilities in adjacent types I, II, III and IV were: 100.00%, 80.00%, 76.00% and 84.78%, respectively. The results show that after incorporating spatial geographic factors, the stability of PHS level in Guangdong Province in type I deteriorates with the increase of adjacency type, and fluctuates and fluctuates with the increase of adjacency type in other types. (3) When the PHS level of neighboring prefecture-level cities increases, the probability of upward transfer of PHS level in local cities can be increased, and after considering the spatial lag factor, the probability of upward transfer will increase when adjacent to prefecture-level cities with higher supply levels. For example, when the observation type is II, with the increase of the adjacent type, the probability of upward transfer were: 26.09%, 16.00%, 41.67% and 46.15%, respectively. These results show that high-level prefecture-level cities have a positive spatial spillover effect on neighboring prefecture-level cities, and have a certain club convergence phenomenon.

**Table 5:** Spatial markov chain transition probability matrix of PHS level in Guangdong Province

Spatial lag	t/t + 1	I	II	III	IV
I	I	0.8448	0.1552	0.0000	0.0000
	II	0.0870	0.6522	0.2609	0.0000
	III	0.0000	0.4286	0.5714	0.0000
	IV	0.0000	0.0000	0.0000	1.0000
II	I	0.7692	0.1923	0.0385	0.000
	II	0.0800	0.7200	0.1600	0.0400
	III	0.0000	0.1250	0.6250	0.2500
	IV	0.0000	0.0000	0.2000	0.8000
III	I	0.4000	0.6000	0.0000	0.0000
	II	0.0417	0.5417	0.4167	0.0000
	III	0.0000	0.1154	0.6154	0.2692
	IV	0.0000	0.0400	0.2000	0.7600
IV	I	0.0000	1.0000	0.0000	0.0000
	II	0.0000	0.5385	0.4615	0.0000
	III	0.0000	0.2857	0.5238	0.1905
	IV	0.0000	0.0217	0.1304	0.8478

### Results of regional difference analysis

#### Spatial differences in public health service provision

**Fig. 8:**
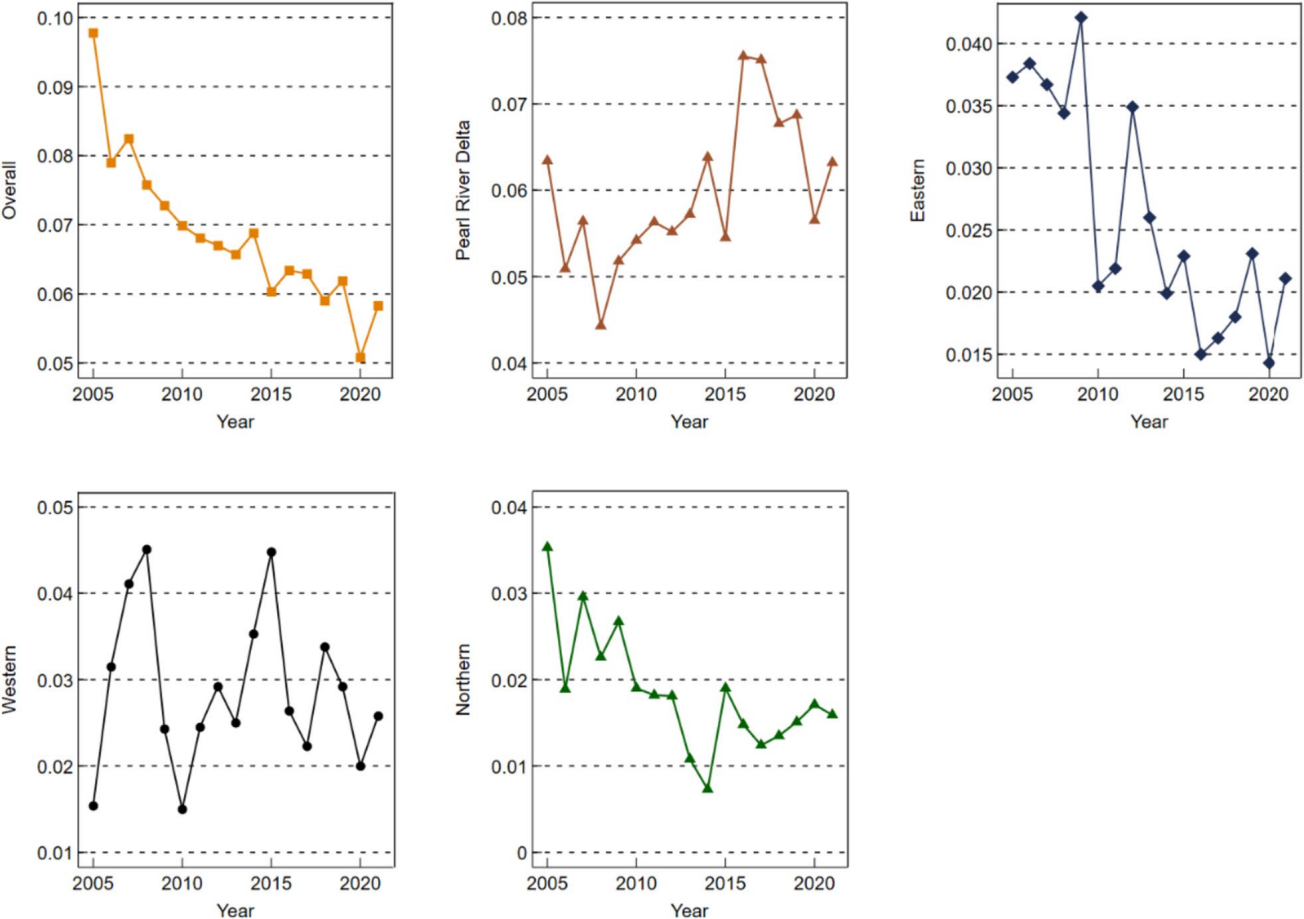
Overall difference and intraregional difference of Gini coefficient of PHS level in Guangdong province

In order to explore the relative difference in PHS levels in Guangdong Province and its sources, this paper calculates and decomposes the regional differences in PHS levels in Guangdong Province from 2005 ~ 2021 according to Dagum’s Gini coefficient, and the results are shown in Appendix Table A2. In order to show the Gini coefficient results more intuitively, we use Fig. 8 ~ 10 to show them. Figure 8 shows the overall and regional differences over the observation period. Among them, the overall Gini coefficient decreased from 0.0978 in 2005 to 0.0583 in 2021, a decrease of 40.39%, indicating that the overall regional difference in PHS level in Guangdong Province in 2005 ~ 2021 continued to narrow, and the equilibrium degree gradually improved. Compared with the obvious downward trend of the overall Gini coefficient, the Gini coefficient in the four major regions fluctuates greatly, for example, the fluctuation of Gini coefficient in the Pearl River Delta region and western Guangdong region is the most prominent, the Gini coefficient fluctuation range in the Pearl River Delta region is 0.0443 ~ 0.0634, and the Gini coefficient fluctuation range in western Guangdong region is 0.0154 ~ 0.0448, and the fluctuation is mainly rising. It showed that the difference in PHS levels between the two regions was still large during the observation period, and the main trend was the increase of the difference. However, the fluctuation and decline trend mainly showed a downward trend in eastern Guangdong and northern Guangdong, with a decrease of 43.43% in eastern Guangdong and 54.96% in northern Guangdong, indicating that the spatial difference in PHS level between eastern Guangdong and northern Guangdong gradually narrowed during the observation period, and the degree of equalization of resource allocation was improved.

**Fig. 9:**
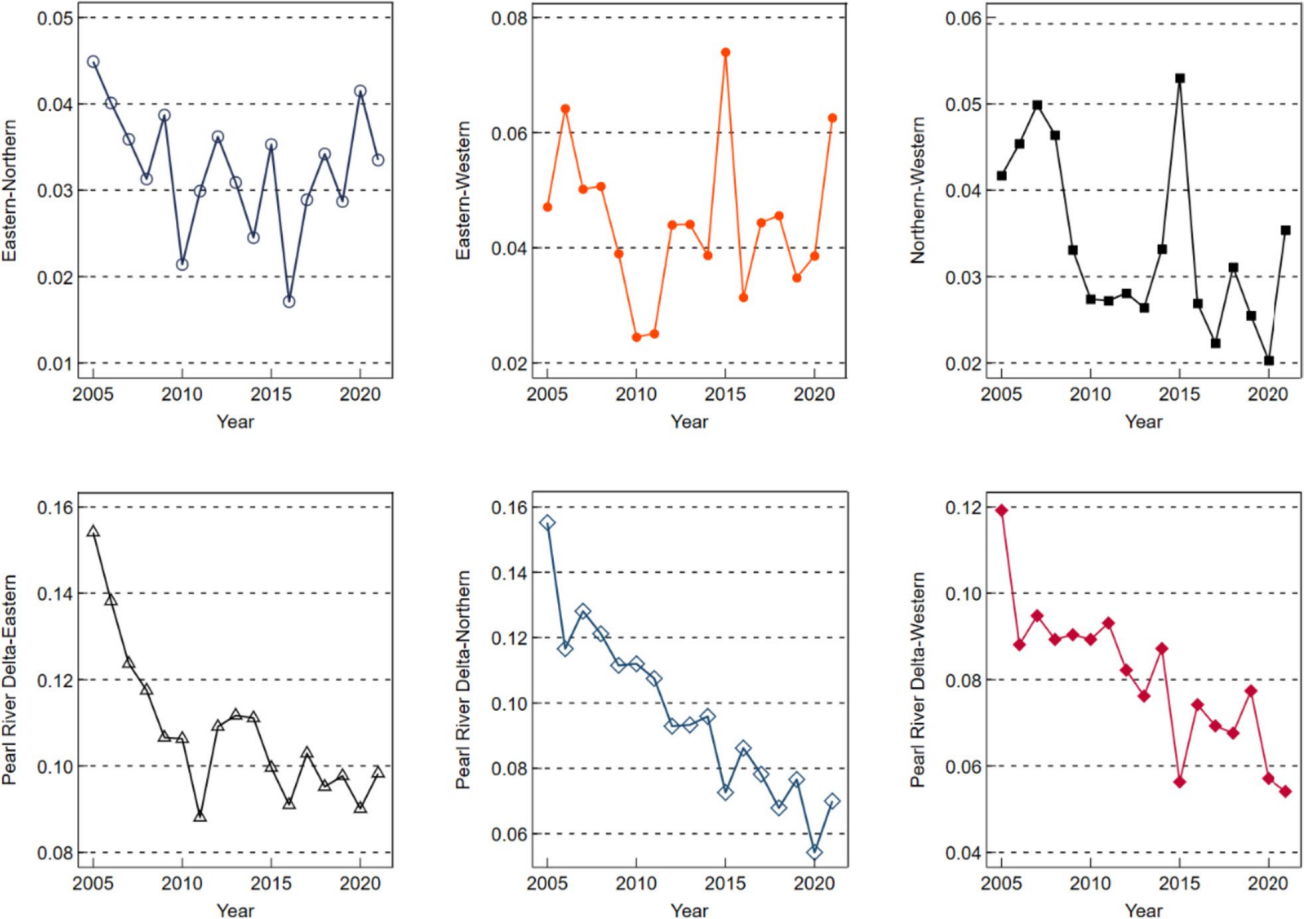
Regional difference of Gini coefficient of PHS level in Guangdong province

Figure 9 shows the difference in Gini coefficient between prefecture-level cities. From the results, the Gini coefficient between various regions in Guangdong Province showed a downward trend in fluctuations on the whole; The Gini coefficients between the Pearl River Delta and eastern Guangdong, the Pearl River Delta and western Guangdong, the Pearl River Delta and northern Guangdong, eastern Guangdong and northern Guangdong, and northern Guangdong and western Guangdong decreased by 36.21%, 54.61%, 54.90%, 25.39% and 15.11%, respectively. It shows that the regional differences between the Pearl River Delta and eastern Guangdong, northern Guangdong and western Guangdong, and between northern Guangdong and eastern Guangdong and western Guangdong have gradually narrowed, the PHS level of the three remote areas of eastern Guangdong, western Guangdong and northern Guangdong has improved, and the difference in resource allocation between them and developed cities is gradually narrowing. However, unfortunately, from Fig. 8, we can also find that the Gini coefficient between eastern Guangdong and western Guangdong shows a fluctuating upward trend, with an increase of 32.91%, indicating that the regional difference between eastern and western Guangdong is gradually widening.

**Fig. 10:**
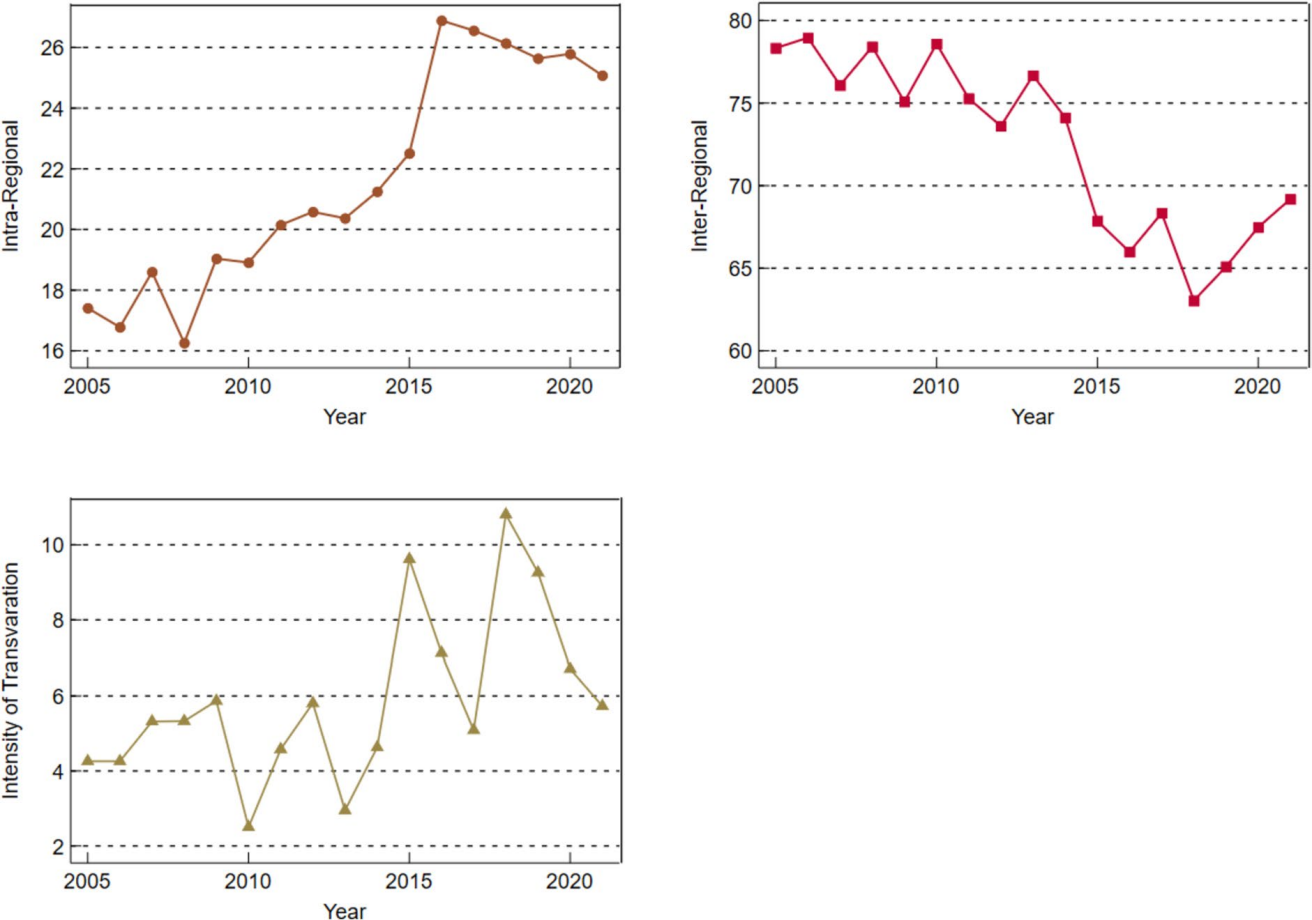
Contribution rate of overall difference of PHS Level in Guangdong province

Figure 10 shows the contribution rate of the overall difference in PHS level in Guangdong Province, including intra-regional contribution rate, inter-regional contribution rate and supervariable density contribution rate, which refers to the impact of cross-overlap between different regions on the overall difference. It can be seen from Fig. 10 that with the increase of years, the interregional contribution rate of the overall difference in PHS level in Guangdong Province fluctuates and decreases; The intra-regional contribution rate and the supervariable density contribution rate both showed a fluctuating upward trend. Among them, the contribution rate accounted for a large proportion of the interregional gap, accounting for more than 63.04%, far exceeding the contribution rate within the region and the contribution rate of over-variable density, indicating that the regional difference in the supply level of public health services in Guangdong Province was mainly caused by the interregional gap. In addition, the contribution rate and supervariable density contribution rate in the region are relatively small, and the proportion of the contribution rate of supervariable density is the lowest, and its highest proportion is only 10.81%, which shows that intraregional differences and cross-overlap between different regions are not the key reasons affecting the overall difference.

#### Analysis of the causes and mechanisms of spatial differences in public health service supply

In order to further explore the key factors affecting the spatial difference of PHS level in Guangdong Province, according to the research idea of Formula (16), referring to the practice of Chen [36], we used STATA.16 software to use random-effects model, fixed-effect model and mixed least squares regression model (OLS) to regression analysis of quantifiable influencing factors, and performed robustness tests to explore the correlation between each factor and PHS level, and the regression results are shown in Table 6. In our paper, to reduce the effect of heteroscedasticity, we uniformly use robustness standard errors in panel regression models. The results of the Breusch-Pagan-Lagrange multiplier test showed that the random-effects model was better than the OLS regression model (statistical coefficient: 645.5100, *P* < 0.001). The results of the F test showed that the random-effects model and the fixed-effect model were better than the OLS regression model (statistical coefficients: 20.6500, *P* < 0.001). The Hausman test showed that the fixed-effect model was superior to the random-effects model (statistical coefficients: 18.4800, *P* = 0.0024 < 0.05). Therefore, we finally decided to use a panel fixed-effect model to analyze the influencing factors of PHS levels in Guangdong Province.

From Table 6, it can be seen that different factors have different effects on PHS levels in Guangdong Province. Firstly, the economic development of prefecture-level cities has a great impact on the local PHS level, with a statistical coefficient of 0.0483 and a t-value of 16.1300, which is statistically significant at the statistical level of 1%, this shows that for every 1% increase in the local economy, the level of public health service provision will increase by 4.83%. Secondly, the degree of fiscal decentralization and urbanization rate both had a positive effect on the PHS level in Guangdong Province (FSS statistical coefficient: 0.0300, t-value: 3.400; UR statistical coefficient: 0.0117, t-value: 1.5600), both of which were significant at the statistical level of 1%. Finally, we can also find that although population factors and the degree of industrial structure upgrading have a positive impact on the PHS level, they are not significant at the statistical level of 5% (LNDEN statistical coefficient: 0.0147, t-value: 1.6500; ISU statistical coefficient: 0.0003, t-value: 1.5600). It shows that compared with other key factors, the effect of regional population density and industrial structure upgrading on the local PHS level is not obvious.

**Table 6:** Regression analysis on the influencing factors of PHS level in Guangdong province

variable	Random effect model	Fixed effect model	OLS model
LNPGDP	0.04837***(17.3100)	0.0483***(16.1300)	0.0437***(7.8400)
FSS	0.0303***(3.6700)	0.0300*** (3.400)	0.0354**(2.3800)
LNDEN	0.0098*(1.8900)	0.0147 (1.6500)	0.0072(1.3200)
UR	0.0112***(2.8700)	0.0117***(2.9800)	0.0068(1.1100)
ISU	0.0004**(2.1500)	0.0003(1.5600)	0.0012(1.6600)
𝛼	-0.2224***(-6.5100)	-0.2487***(-4.8400)	-0.1929***(-3.3600)
*R* ^*2*^	0.8045	0.7919	0.7784
*N*	357	357	357
*F*		133.0600***[0.0000]	39.27***[0.0000]
Wald chi^2^	759.78***[0.0000]		
B-P LM test(chi^2^)			645.51***[0.0000]
*F*(all u_i = 0)		20.6500***[0.0000]	
Hausman test	18.4800**[0.0024]		

*P < 0.1, **P < 0.05, ***P < 0.01, α is the constant term, *N* is the number of samples, values in the square brackets are the *P* value, and values in the bracket are the t value

We further analyze the mechanisms by which factors with significant effects influence the level of public health service provision. Hansen [47] obtains its asymptotic distribution through the use of the “Bootstrap method”, thereby constructing its P-value. The number of thresholds is determined by observing the F-statistics and P-values in Table 7. Considering the results of the single threshold, double threshold, and triple threshold effect models, we find that both the urbanization rate and the degree of fiscal decentralization have statistically significant single threshold effects (*F*_*UR*_=60.7300, *P*_*UR*_=0.0267; *F*_*FSS*_=152.4600, *P*_*FSS*_<0.001) as well as double threshold effects (*F*_*UR*_=35.8300, *P*_*UR*_=0.0467; *F*_*FSS*_=35.6400, *P*_*FSS*_=0.0333).

**Table 7:** Significance tests of threshold variables as well as threshold value estimates

The threshold effect	UR			FSS		
	F-value	*P*-value	Number of BS	F-value	*P*-value	Number of BS
A single threshold	60.7300	0.0267	300	152.4600	<0.001	300
Double threshold	35.8300	0.0467	300	36.5400	0.0333	300
Triple threshold	40.7100	0.9367	300	41.3300	0.7800	300

The *P*-value is the result obtained by repeated sampling using the “self-sampling method” (Bootstrap)

The results of the threshold regression are shown in Table 8. Under different threshold conditions, (1) from the trend of changes in the estimated coefficients of the threshold interval, the impact of the urbanization rate on the PHS level gradually increases, with threshold values of 9.7638 and 11.5076 respectively. When the level of regional economic development is less than the threshold value, the influence coefficient is 0.0359 (*P* < 0.001); when the level of economic development exceeds the threshold value, the influence coefficient is 0.0687 (*P* < 0.001). (2) The effect of the degree of fiscal decentralization on the PHS level gradually increases, with threshold values of 9.8257 and 11.4208 respectively. When the level of economic development is less than the threshold value, the influence coefficient is 0.0484 (*P* < 0.001); when the level of economic development crosses the threshold value, the influence coefficient is the influence coefficient is 0.0831 (*P* < 0.001). The aforementioned results indicate that with the improvement of the regional economic development level, high urbanization rates and high fiscal decentralization levels in different cities will contribute to the enhancement of their PHS levels.

**Table 8:** Threshold model regression results

Variable	UR		FSS	
Threshold value	9.7638, 11.5076		9.8257, 11.4208	
	estimated value	95% confidence interval	estimated value	95% confidence interval
γ_1_	0.0359***	(0.0276, 0.0443)		
γ_2_	0.0687***	(0.0546, 0.0828)		
γ’_1_			0.0484***	(0.0292, 0.0676)
γ’_2_			0.0831***	(0.0620, 0.1043)
Control variable	Control	Control	Control	Control
*F*	23.3600	23.3600	14.0600	14.0600
*R* ^*2*^	0.6170	0.6170	0.6578	0.6578

*P < 0.1, **P < 0.05, ***P < 0.01

## Discussion

This is a regional study on the spatiotemporal characteristics of PHS levels in Guangdong Province. Previous studies on the spatial distribution of medical resources: At the national level, these works focus more on macro studies in 31 provinces [58], at the non-national level, they focus more on the research of a megacity [59], and a few literature studies on the allocation of medical resources in one province [60]. However, the above studies still lack the spatial correlation and heterogeneity of public health service provision in different levels of prefecture-level cities within the province. Therefore, our paper analyzes the dynamic evolution and spatial differences and influencing factors of public health service supply levels in 21 prefecture-level cities in Guangdong Province from 2005 ~ 2021 by combining standard deviation ellipse method, kernel density estimation, Markov chain model, Gini coefficient and panel regression model. In addition, the threshold effect model was used to further analyze the underlying mechanisms. The results of the study showed that:

(1)From the perspective of spatial distribution and centroid migration results, the PHS level in Guangdong Province generally presents an upward spatiotemporal evolution trend, with the centroid shifting from the southwest to the northeast. Moreover, the PHS level in peripheral cities such as those in the southwest or northeast regions is rising at a faster rate. (2) From the dynamic characteristics analysis of the kernel density estimation, the overall supply level of public health services in Guangdong Province is gradually improving. However, there is a polarization phenomenon and a gradient effect in the Pearl River Delta and eastern Guangdong regions. (3) From the dynamic characteristic analysis of the Markov chain, the supply level of public health services in Guangdong Province has been generally evolving towards a higher level during the observation period, with a relatively small probability of leapfrog transitions. Prefecture-level cities with higher supply levels have a positive spillover effect, showing a certain phenomenon of club convergence. (4)From the analysis of spatial differences, influencing factors, and mechanisms, the overall differences, intra-regional differences, and inter-regional differences in the PHS level in Guangdong Province have different evolution trends during the observation period. Spatial differences still exist, mainly between regions. These differences are positively affected more significantly by factors such as the level of regional economic development, the degree of fiscal decentralization, and the urbanization rate. Moreover, the indirect effects of the level of economic development on the PHS level through the urbanization rate and the degree of fiscal decentralization both exhibit a double threshold effect.

In China, it is undisputed that the economically developed provinces along the eastern coast have access to superior medical resources [61]. Often, the provision of basic public services is related to regional resource endowment [62]. Due to the good geographical accessibility and outstanding advantages, the public health service supply level in the Pearl River Delta region is at a high level, but there has always been a contradiction of uncoordinated development within urban agglomerations [60, 63]. This is not surprising, as economic opportunities are generally considered the main determinant of population mobility [64]. Since the implementation of the reform and opening-up policies in 1979, Guangdong Province has been home to the first and second batches of pilot cities for these reforms, such as Shenzhen, Zhuhai, Shantou, Guangzhou, Zhanjiang, etc. These pilot cities have experienced rapid socio-economic development, creating new momentum and attraction for nationwide population mobility seeking employment opportunities in Guangdong [65]. The new medical reform policy in 2009, namely the “Opinions on Deepening the Reform of the Medical and Health System,” encouraged local governments to improve public health service projects in line with their actual economic development level, aiming to achieve a balanced development of public health service levels within the region. As the province with the largest economy and population in China, the Guangdong provincial government has consistently strived to link the social mission of building a healthy province with the goal of sustainable socio-economic development [60].

For instance, on one hand, the provincial government has proposed to build a county-level medical community model that provides high-quality services, carries out collaborative division of labor between the “headquarters and branches,” and operates effectively, to better complete the construction of a tiered diagnosis and treatment system. The construction of a tiered diagnosis and treatment system contributes to optimizing the allocation of public health resources in Guangdong Province, making better use of existing health resources to serve the basic public health needs of residents, and promoting the equalization of basic public health services [66]. These systems are never a single institutional design or reform task; they are the natural result of effective medical reform and the inevitable state of a high-quality and efficient medical service system. Therefore, it is necessary to deepen the reform of the medical system comprehensively. The Guangdong provincial government has proposed a series of key medical reform tasks in line with reality, creating a favorable policy environment for the improvement of the public health service level in various prefecture-level cities. On the other hand, the governments of remote areas have actively learned from the “Luohu Model” (the main direction of urban medical system reform) and the “Huadu Model” (a model example of grassroots medical system reform) in the Pearl River Delta region to develop local public health services [67]. They have also implemented systems such as “Category I Public Welfare Guarantee, Category II Public Welfare Performance Management” within the region to attract high-quality medical resources to the grassroots level. On the whole, after years of exploration and summarization of experiences, Guangdong Province has achieved significant improvements in the allocation of public health resources.

However, it is regrettable that in Guangdong Province, which ranks among the top in economic strength and has a relatively abundant overall level of medical supply, there still exist prominent issues of spatial allocation imbalance and mismatch of supply and demand. This has also been confirmed in previous studies [63]. The regional disparities in basic public services have become a universal issue worldwide due to unequal opportunities to access these services [68]. While maintaining the current overall level of public health service supply, how to discern the key factors causing these disparities in order to better reduce the supply differences of public health services within regions, is a challenge we are currently facing and may continue to face in the long term. Some studies have discussed the relationship between regional economic development and public service supply [69]: regions with higher incomes and higher economic levels have stronger fiscal extraction and disposable capacity of local governments, and they can provide more public services for residents [70, 71]. Our conclusions on influencing factors have once again validated this perspective. Furthermore, the threshold effect analysis suggests that re-examining the indirect effects of urbanization rate and fiscal decentralization on the level of public health service supply under different economic development conditions, holds significant practical implications for targeted allocation of public health services.

### Conclusions and recommendations

Paying attention to the current status and influencing mechanisms of public health service supply, and exploring strategies for its spatial allocation optimization, is a key approach to addressing the imbalance in public health services. This paper discusses the dynamic evolution and spatial differences in the level of public health service supply in prefecture-level cities in Guangdong Province. It was found that the overall level of PHS is on the rise, but there still exist spatial differences at the prefecture-level city scale, mainly between regions. This further enriches the research scope on the balance of public health resource allocation between regions within economically developed provinces. Furthermore, unlike previous studies that solely analyzed influencing factors, we further condensed the paths of action between key factors affecting these differences based on preliminary research, and found that both urbanization rate and fiscal decentralization have a dual threshold effect on the PHS level. This provides a reference for the scientific formulation of public health service policies. Based on the above conclusions, we can consider that improvements can be made in the following aspects: (1) Implement public health resource allocation according to local conditions. Empirical results corroborate that the overall level of public health service supply in Guangdong Province is gradually improving, but regional differences still persist. Therefore, it is necessary to deepen the structural reform of the supply side of medical and health services. A specific coordinating body could be established, responsible for formulating, implementing, and supervising policy documents in various regions, dynamically adjusting policies according to local conditions to prevent the widening of inter-regional differences. Additionally, it is necessary to establish a comprehensive medical and health service system based on fundamental conditions such as population size and actual demand, plan the layout of limited public health resources scientifically, and ensure that populations in different regions can all fairly obtain the public health services they actually need. (2)The government should pay full attention to the spatial linkage between prefecture-level cities and enhance the positive spillover effect in areas with high supply capacity. 2022 is a new starting point for medical innovation in Guangdong Province, the whole country and even the world, under the influence of multiple variables such as policy promotion, technology iteration, and the impact of the epidemic, it is necessary for developed and underdeveloped regions to fully utilize modern information technology to build a health resource interactive platform under the concept of “resource sharing”. Establishing an “Internet + Medical” model, deeply integrating the Industrial 4.0 revolution with Health 4.0, it aims to achieve benign exchanges and cooperation between developed and underdeveloped areas in terms of technology, talent, and medical achievements, fully playing the radiating and driving role of regions with a high level of public health service supply. (3) Emphasis should be placed on enhancing the “hematopoietic” driving elements in underdeveloped areas, to avoid over-reliance on external “transfusion” support. While considering the radiating and driving basis of developed regions, underdeveloped areas should focus on the excavation and enhancement of their own “hematopoietic” functional elements. Regular research and analysis on the supply status and its reasons in both local and developed areas should be conducted to identify strengths and weaknesses. Efforts should be made to actively maintain advantageous supplies, weaken or even eliminate the hindrance of disadvantages. This approach fundamentally enhances their own service supply capabilities, truly narrowing the supply gap with developed areas. (4) Full consideration should be given to the threshold characteristics of key factors, promoting the spatial adaptability of urbanization, fiscal decentralization, and the level of public health service supply. Firstly, the promotion of a new type of urbanization in China should be actively and prudently advanced. For instance, economically developed regions need to continue leveraging their resource and location advantages, incorporating the quality and sustainable development of public health service supply into their assessment and incentive scope, thereby forming a “benchmark effect” among regions. As for underdeveloped areas, they need to manage the relationship between government and the market effectively, allowing market mechanisms to play a decisive role in resource allocation, activating the endogenous power of economic growth, forming a beneficial “competition effect” in resource allocation among regions, enabling their economic development level to reach above the “threshold” as soon as possible, ultimately contributing to further improvement in public health service supply capacity. Secondly, the fiscal decentralization system should be improved and the regional performance assessment system should be enhanced. A centralization and decentralization system with clear rights and responsibilities should be established, appropriately distributing the management scope of governments at all levels. With economic development, the autonomy of local governments in fiscal fund allocation should be constantly adjusted and optimized, enabling them to achieve the goal of promoting equal development of local public health resource allocation.

## Future research and deficiencies

This study explores the dynamic evolution, spatial differences, and influencing factors of the public health service supply level in the prefecture-level cities of Guangdong Province. It further condenses the action mechanism of the key factors, thereby enriching the research scope of the balance of public health resource allocation between regions within the province. However, this article also has the following limitations: (1) In view of the current regulations on public health service projects, our paper incorporates health education indicators into the system for evaluation, which is expected to evaluate the public health service supply level in Guangdong Province more scientifically and objectively. However, due to the availability of data, only the indicator of “number of health examinations” is currently included, and indicators in other public health service projects, such as “printed materials on health education” and “number of public participations in health education activities”, cannot be collected, and the evaluation index system of the scope of public health services should be further enriched in future research. (2) At present, our research mainly discusses spatial differences from the key influencing factors and their mechanisms of the supply side, and the influence of the demand side should also be considered in future research, so as to evaluate the balance of public health service supply in Guangdong Province from the perspective of supply and demand more scientifically and objectively.

### Supplementary Information

**Additional file 1: Appendix A. Table A.1. **The Guangdong’s medical and health services (PHS) level from 2005- 2021. **Table A.2. **Gini coefficient and decomposition from 2005 to 2021.

## Data Availability

The original data can be queried on the official website of Guangdong Provincial Health Commission(http://wsjkw.gd.gov.cn/) and Guangdong Statistical Information Network (http://stats.gd.gov.cn/), and the Resource and Environment Data Cloud Platform (http://www.resdc.cn). Further inquiries can be directed to the corresponding author.
