# Supplementary material for: Dynamic evolution and spatial difference of public health service supply in economically developed provinces of China: typical evidence from Guangdong Province
Source: BMC Health Serv Res. 2024 Jan 4;24:23. doi: 10.1186/s12913-023-10444-4 (PMC10768127; doi:10.1186/s12913-023-10444-4)
Supplement: Supplementary file 1 — Additional file 1: Appendix A. Table A.1. The Guangdong’s medical and health services (PHS) level from 2005- 2021. Table A.2. Gini coefficient and decomposition from 2005 to 2021. [file 12913_2023_10444_MOESM1_ESM.docx]

**Appendix A**

**Table A.1**

The Guangdong’s medical and health services (PHS) level from 2005- 2021

| Regions | city | The Guangdong’s medical and health services (PHS) level | | | | | | | | | | | | | | | | |
| --- | --- | --- | --- | --- | --- | --- | --- | --- | --- | --- | --- | --- | --- | --- | --- | --- | --- | --- |
|  |  | 2  0  0  5 | 2006 | 2007 | 2008 | 2009 | 2010 | 2011 | 2012 | 2013 | 2014 | 2015 | 2016 | 2017 | 2018 | 2019 | 2020 | 2  0  2  1 |
| Pearl River Delta | Guangzhou | 0.  4  2  5  3 | 0.4514 | 0.4757 | 0.4726 | 0.4984 | 0.5084 | 0.5290 | 0.5518 | 0.5639 | 0.5854 | 0.5744 | 0.5957 | 0.5939 | 0.5566 | 0.5703 | 0.5241 | 0.5  6  1  1 |
|  | Shenzhen | 0.  4  6  2  6 | 0.3974 | 0.4285 | 0.4396 | 0.4526 | 0.4670 | 0.4781 | 0.4718 | 0.4807 | 0.4931 | 0.4994 | 0.4814 | 0.5071 | 0.4950 | 0.5112 | 0.4923 | 0.5  1  8  1 |
|  | Zhuhai | 0.  4  0  4  2 | 0.3965 | 0.4087 | 0.4242 | 0.4074 | 0.4074 | 0.4219 | 0.4274 | 0.4304 | 0.4412 | 0.4471 | 0.4124 | 0.4224 | 0.4246 | 0.4394 | 0.4157 | 0.4  2  4  3 |
|  | Foshan | 0.  4  0  6  5 | 0.3828 | 0.4089 | 0.4150 | 0.4178 | 0.4291 | 0.4395 | 0.4550 | 0.4748 | 0.4910 | 0.4897 | 0.4747 | 0.4847 | 0.4802 | 0.4836 | 0.4512 | 0.4  6  6  1 |
|  | Huizhou | 0.  3  4  8  0 | 0.3666 | 0.3687 | 0.3915 | 0.3729 | 0.3751 | 0.3992 | 0.4044 | 0.4138 | 0.4147 | 0.4322 | 0.3969 | 0.4080 | 0.3996 | 0.4073 | 0.3871 | 0.4  1  1  8 |
|  | Dongguang | 0.  4  3  0  3 | 0.3990 | 0.4186 | 0.4006 | 0.4385 | 0.4437 | 0.4340 | 0.4496 | 0.4556 | 0.4464 | 0.4504 | 0.4337 | 0.4477 | 0.4461 | 0.4601 | 0.4345 | 0.4  5  2  6 |
|  | Zhongshan | 0.  4  1  3  2 | 0.4195 | 0.4233 | 0.4144 | 0.4245 | 0.4296 | 0.4330 | 0.4322 | 0.4379 | 0.4351 | 0.4403 | 0.4285 | 0.4264 | 0.4168 | 0.4267 | 0.4136 | 0.4  1  5  3 |
|  | Jiangmen | 0.  3  6  0  8 | 0.3584 | 0.3533 | 0.3972 | 0.3982 | 0.4108 | 0.4164 | 0.4244 | 0.4165 | 0.4255 | 0.4444 | 0.4052 | 0.4029 | 0.4133 | 0.4245 | 0.3967 | 0.4  1  1  8 |
|  | Zhaoqing | 0.  3  0  5  1 | 0.3171 | 0.3324 | 0.3415 | 0.3627 | 0.3664 | 0.3554 | 0.3754 | 0.3884 | 0.3842 | 0.3950 | 0.3604 | 0.3731 | 0.3646 | 0.3700 | 0.3876 | 0.3  8  9  4 |
|  | Average | 0.  3  9  5  1 | 0.3876 | 0.4020 | 0.4107 | 0.4192 | 0.4264 | 0.4341 | 0.4436 | 0.4513 | 0.4574 | 0.4637 | 0.4432 | 0.4518 | 0.4441 | 0.4548 | 0.4337 | 0.4  5  0  1 |
| Eastern Guangdong province | Shantou | 0.  3  2  2  3 | 0.3229 | 0.3396 | 0.3486 | 0.3694 | 0.3592 | 0.3727 | 0.3911 | 0.3879 | 0.3878 | 0.4062 | 0.3877 | 0.3828 | 0.3830 | 0.3944 | 0.3743 | 0.3  9  1  1 |
|  | Shanwei | 0.  2  7  6  4 | 0.2721 | 0.2877 | 0.2984 | 0.3052 | 0.3274 | 0.3819 | 0.3349 | 0.3483 | 0.3557 | 0.3671 | 0.3644 | 0.3557 | 0.3531 | 0.3554 | 0.3510 | 0.3  5  6  0 |
|  | Chaozhou | 0.  2  7  3  4 | 0.2866 | 0.3147 | 0.3275 | 0.3421 | 0.3468 | 0.3457 | 0.3461 | 0.3456 | 0.3549 | 0.3681 | 0.3627 | 0.3658 | 0.3691 | 0.3784 | 0.3607 | 0.3  6  1  8 |
|  | Jieyang | 0.  2  9  8  9 | 0.3082 | 0.3272 | 0.3268 | 0.3478 | 0.3443 | 0.3421 | 0.3599 | 0.3625 | 0.3683 | 0.3842 | 0.3669 | 0.3806 | 0.3823 | 0.3855 | 0.3776 | 0.3  8  4  9 |
|  | Average | 0.  2  9  2  7 | 0.2974 | 0.3173 | 0.3253 | 0.3411 | 0.3444 | 0.3606 | 0.3580 | 0.3611 | 0.3667 | 0.3814 | 0.3704 | 0.3712 | 0.3719 | 0.3784 | 0.3659 | 0.3  7  3  5 |
| Western Guangdong province | Yangjiang | 0.  3  0  0  7 | 0.2985 | 0.3313 | 0.3191 | 0.3294 | 0.3414 | 0.3412 | 0.3548 | 0.3669 | 0.3532 | 0.3875 | 0.3714 | 0.3852 | 0.3660 | 0.3719 | 0.3751 | 0.4  1  7  9 |
|  | Zhanjiang | 0.  3  1  5  2 | 0.3451 | 0.3332 | 0.3313 | 0.3530 | 0.3626 | 0.3652 | 0.3754 | 0.3895 | 0.3920 | 0.4472 | 0.3839 | 0.3962 | 0.4041 | 0.3919 | 0.3877 | 0.3  9  5  1 |
|  | Maoming | 0.  3  2  2  3 | 0.3417 | 0.3683 | 0.3895 | 0.3678 | 0.3654 | 0.3812 | 0.4045 | 0.4106 | 0.4147 | 0.4756 | 0.4178 | 0.4256 | 0.4266 | 0.4240 | 0.4103 | 0.4  4  4  0 |
|  | Average | 0.  3  1  2  8 | 0.3284 | 0.3359 | 0.3466 | 0.3501 | 0.3565 | 0.3625 | 0.3782 | 0.3890 | 0.3866 | 0.4368 | 0.3910 | 0.4023 | 0.3989 | 0.3959 | 0.3910 | 0.3  9  1  0 |
| Northern Guangdong province | Shaoguan | 0.  2  6  4  2 | 0.2828 | 0.2869 | 0.3029 | 0.3088 | 0.3197 | 0.3345 | 0.3450 | 0.3668 | 0.3777 | 0.3970 | 0.3621 | 0.3844 | 0.3896 | 0.3852 | 0.3855 | 0.3  9  2  2 |
|  | Heyuan | 0.  2  9  6  3 | 0.3104 | 0.3222 | 0.3284 | 0.3484 | 0.3540 | 0.3680 | 0.3759 | 0.3775 | 0.3802 | 0.4307 | 0.3758 | 0.3871 | 0.3886 | 0.3991 | 0.3975 | 0.3  9  6  3 |
|  | Meizhou | 0.  2  6  0  8 | 0.3076 | 0.2891 | 0.3045 | 0.3146 | 0.3296 | 0.3434 | 0.3784 | 0.3782 | 0.3753 | 0.3954 | 0.3895 | 0.3834 | 0.4148 | 0.4198 | 0.4164 | 0.4  1  6  3 |
|  | Qingyuan | 0.  3  0  5  7 | 0.3227 | 0.3109 | 0.3285 | 0.3397 | 0.3458 | 0.3593 | 0.3810 | 0.3846 | 0.3853 | 0.4022 | 0.3877 | 0.4071 | 0.3943 | 0.3939 | 0.3974 | 0.3  8  7  9 |
|  | Yunfu | 0.  3  0  8  7 | 0.3095 | 0.3281 | 0.3405 | 0.3513 | 0.3494 | 0.3545 | 0.3706 | 0.3761 | 0.3775 | 0.4082 | 0.3747 | 0.3963 | 0.3968 | 0.3918 | 0.3857 | 0.3  7  9  1 |
|  | Average | 0.  2  8  7  1 | 0.3066 | 0.3074 | 0.3210 | 0.3326 | 0.3397 | 0.3520 | 0.3702 | 0.3766 | 0.3792 | 0.4067 | 0.3779 | 0.3916 | 0.3968 | 0.3980 | 0.3965 | 0.3  9  4  4 |

**Table A.2**

Gini coefficient and decomposition from 2005 to 2021

| Year | Overall | Intra-Regional | | | | Inter-Regional | | | | | | Contribution Rate（%） | | |
| --- | --- | --- | --- | --- | --- | --- | --- | --- | --- | --- | --- | --- | --- | --- |
|  |  | Pearl River Delta | Eastern | Western | Northern | Pearl River Delta-Eastern | Pearl River Delta-Western | Pearl River Delta-Northern | Eastern-Western | Eastern-Northern | Northern-Western | Intra-Regional | Inter-Regional | Intensity of Transvaration |
| 2005 | 0.0978 | 0.0634 | 0.0373 | 0.0154 | 0.0353 | 0.1541 | 0.1192 | 0.1552 | 0.0471 | 0.0449 | 0.0417 | 17.41 | 78.33 | 4.26 |
| 2006 | 0.0790 | 0.0509 | 0.0384 | 0.0315 | 0.0189 | 0.1382 | 0.0881 | 0.1166 | 0.0642 | 0.0401 | 0.0454 | 16.78 | 78.96 | 4.26 |
| 2007 | 0.0825 | 0.0564 | 0.0367 | 0.0411 | 0.0296 | 0.1237 | 0.0948 | 0.1281 | 0.0502 | 0.0359 | 0.0499 | 18.60 | 76.08 | 5.32 |
| 2008 | 0.0758 | 0.0443 | 0.0344 | 0.0451 | 0.0226 | 0.1175 | 0.0893 | 0.1212 | 0.0507 | 0.0313 | 0.0464 | 16.26 | 78.41 | 5.33 |
| 2009 | 0.0728 | 0.0518 | 0.0421 | 0.0243 | 0.0267 | 0.1066 | 0.0904 | 0.1115 | 0.0390 | 0.0387 | 0.0331 | 19.04 | 75.09 | 5.87 |
| 2010 | 0.0699 | 0.0542 | 0.0205 | 0.0150 | 0.0190 | 0.1063 | 0.0893 | 0.1120 | 0.0245 | 0.0214 | 0.0274 | 18.91 | 78.59 | 2.50 |
| 2011 | 0.0681 | 0.0563 | 0.0219 | 0.0245 | 0.0182 | 0.0881 | 0.0931 | 0.1075 | 0.0251 | 0.0299 | 0.0272 | 20.15 | 75.28 | 4.58 |
| 2012 | 0.0670 | 0.0552 | 0.0349 | 0.0292 | 0.0181 | 0.1091 | 0.0822 | 0.0929 | 0.0440 | 0.0362 | 0.0281 | 20.58 | 73.61 | 5.81 |
| 2013 | 0.0657 | 0.0572 | 0.0260 | 0.0250 | 0.0108 | 0.1117 | 0.0762 | 0.0933 | 0.0441 | 0.0309 | 0.0264 | 20.37 | 76.67 | 2.95 |
| 2014 | 0.0688 | 0.0638 | 0.0199 | 0.0353 | 0.0073 | 0.1111 | 0.0872 | 0.0959 | 0.0387 | 0.0245 | 0.0332 | 21.25 | 74.12 | 4.63 |
| 2015 | 0.0603 | 0.0545 | 0.0229 | 0.0448 | 0.0190 | 0.0996 | 0.0563 | 0.0726 | 0.0740 | 0.0353 | 0.0530 | 22.51 | 67.87 | 9.62 |
| 2016 | 0.0634 | 0.0755 | 0.0150 | 0.0264 | 0.0148 | 0.0910 | 0.0742 | 0.0862 | 0.0314 | 0.0171 | 0.0269 | 26.89 | 65.99 | 7.12 |
| 2017 | 0.0629 | 0.0751 | 0.0163 | 0.0223 | 0.0124 | 0.1029 | 0.0693 | 0.0782 | 0.0444 | 0.0289 | 0.0223 | 26.56 | 68.34 | 5.09 |
| 2018 | 0.0590 | 0.0677 | 0.0180 | 0.0338 | 0.0135 | 0.0952 | 0.0676 | 0.0679 | 0.0456 | 0.0342 | 0.0311 | 26.14 | 63.04 | 10.81 |
| 2019 | 0.0619 | 0.0687 | 0.0231 | 0.0292 | 0.0151 | 0.0977 | 0.0774 | 0.0766 | 0.0348 | 0.0287 | 0.0255 | 25.64 | 65.10 | 9.26 |
| 2020 | 0.0508 | 0.0565 | 0.0143 | 0.0200 | 0.0171 | 0.0901 | 0.0571 | 0.0543 | 0.0386 | 0.0415 | 0.0203 | 25.79 | 67.48 | 6.72 |
| 2021 | 0.0583 | 0.0632 | 0.0211 | 0.0258 | 0.0159 | 0.0983 | 0.0541 | 0.0700 | 0.0626 | 0.0335 | 0.0354 | 25.08 | 69.19 | 5.73 |
